# Author Correction: Split intein-mediated selection of cells containing two plasmids using a single antibiotic

**DOI:** 10.1038/s41467-019-13716-y

**Published:** 2020-01-09

**Authors:** Navaneethan Palanisamy, Anna Degen, Anna Morath, Jara Ballestin Ballestin, Claudia Juraske, Mehmet Ali Öztürk, Georg A. Sprenger, Jung-Won Youn, Wolfgang W. Schamel, Barbara Di Ventura

**Affiliations:** 1 Signalling Research Centres BIOSS and CIBSS, University of Freiburg, 79104 Freiburg, Germany; 2 Institute of Biology II, Faculty of Biology, University of Freiburg, 79104 Freiburg, Germany; 3 Heidelberg Biosciences International Graduate School (HBIGS), 69120 Heidelberg, Germany; 4 BioQuant Center for Quantitative Biology, University of Heidelberg, 69120 Heidelberg, Germany; 5 DKFZ Graduate School, University of Heidelberg, 69120 Heidelberg, Germany; 6 Department of Immunology, Institute of Biology III, Faculty of Biology, University of Freiburg, 79104 Freiburg, Germany; 7 Spemann Graduate School of Biology and Medicine (SGBM), University of Freiburg, 79104 Freiburg, Germany; 8 Center for Chronic Immunodeficiency (CCI), Medical Center Freiburg and Faculty of Medicine, University of Freiburg, 79104 Freiburg, Germany; 9 Institute of Microbiology, University of Stuttgart, 70569 Stuttgart, Germany

**Keywords:** Genetic engineering, Metabolic engineering, Computational biology and bioinformatics

Correction to: *Nature Communications* 10.1038/s41467-019-12911-1, published online 31 October 2019.

The original version of this Article contained an error in Fig. 7. In panels c and d, the *y*-axes for L-PAPA concentration in mM were incorrectly labelled as nM. This has been corrected in both the PDF and HTML versions of the Article.

