# Supplementary material for: Split intein-mediated selection of cells containing two plasmids using a single antibiotic
Source: Nat Commun. 2019 Oct 31;10:4967. doi: 10.1038/s41467-019-12911-1 (PMC6823396; doi:10.1038/s41467-019-12911-1)
Supplement: Supplementary file 1 — Supplementary Information [file 41467_2019_12911_MOESM1_ESM.pdf]

Split intein-mediated selection of cells containing two plasmids using a single antibiotic

Palanisamy *et al.*

Table of contents:

1) Supplementary Figures 1 – 26
2) Supplementary Note 1
3) Supplementary Table 1
4) References

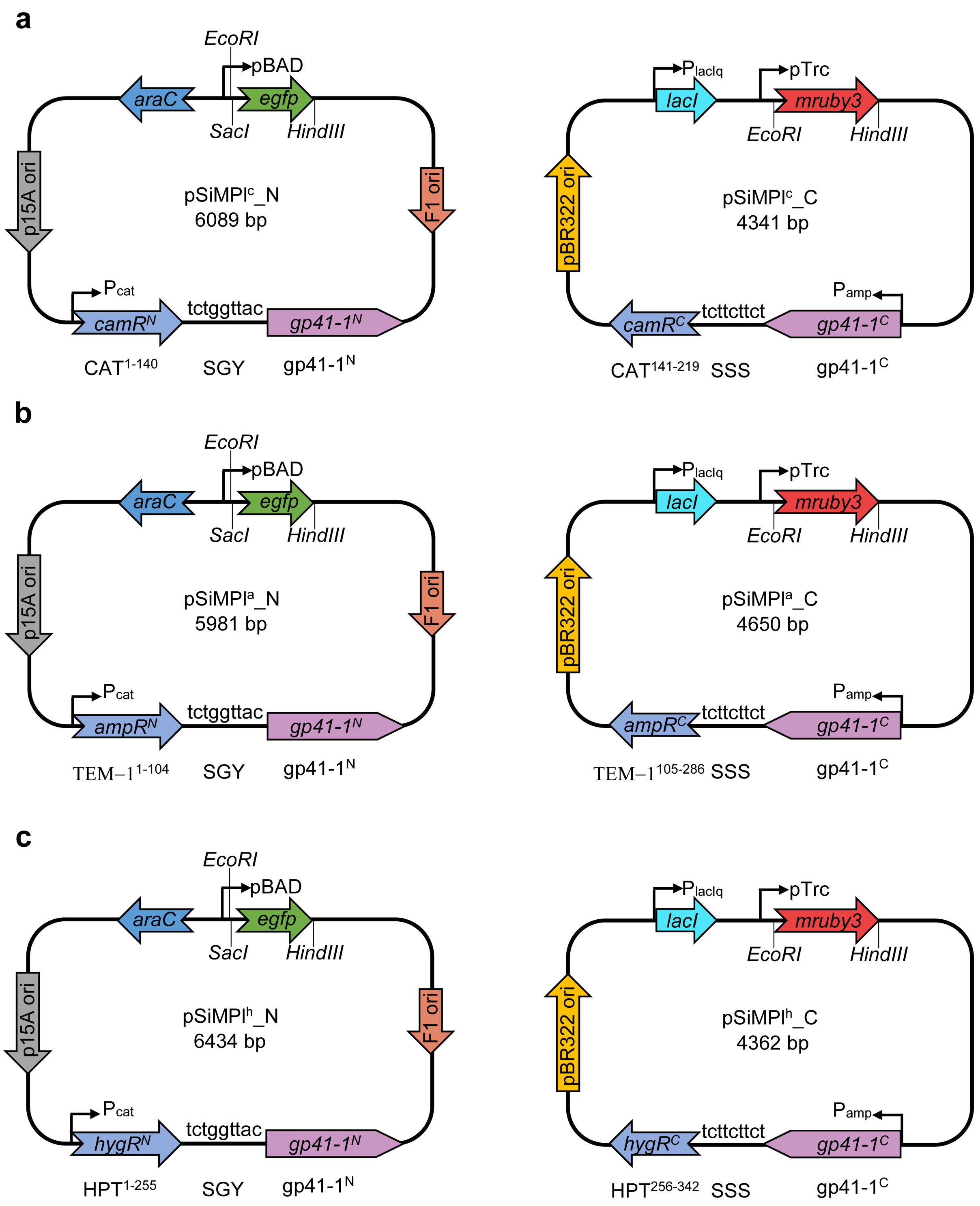

Supplementary Fig. 1 The SiMPl toolkit for use in bacteria. Schematic showing the main features found on the SiMPl plasmids for use with chloramphenicol (**a**), ampicillin (**b**) and hygromycin (**c**).

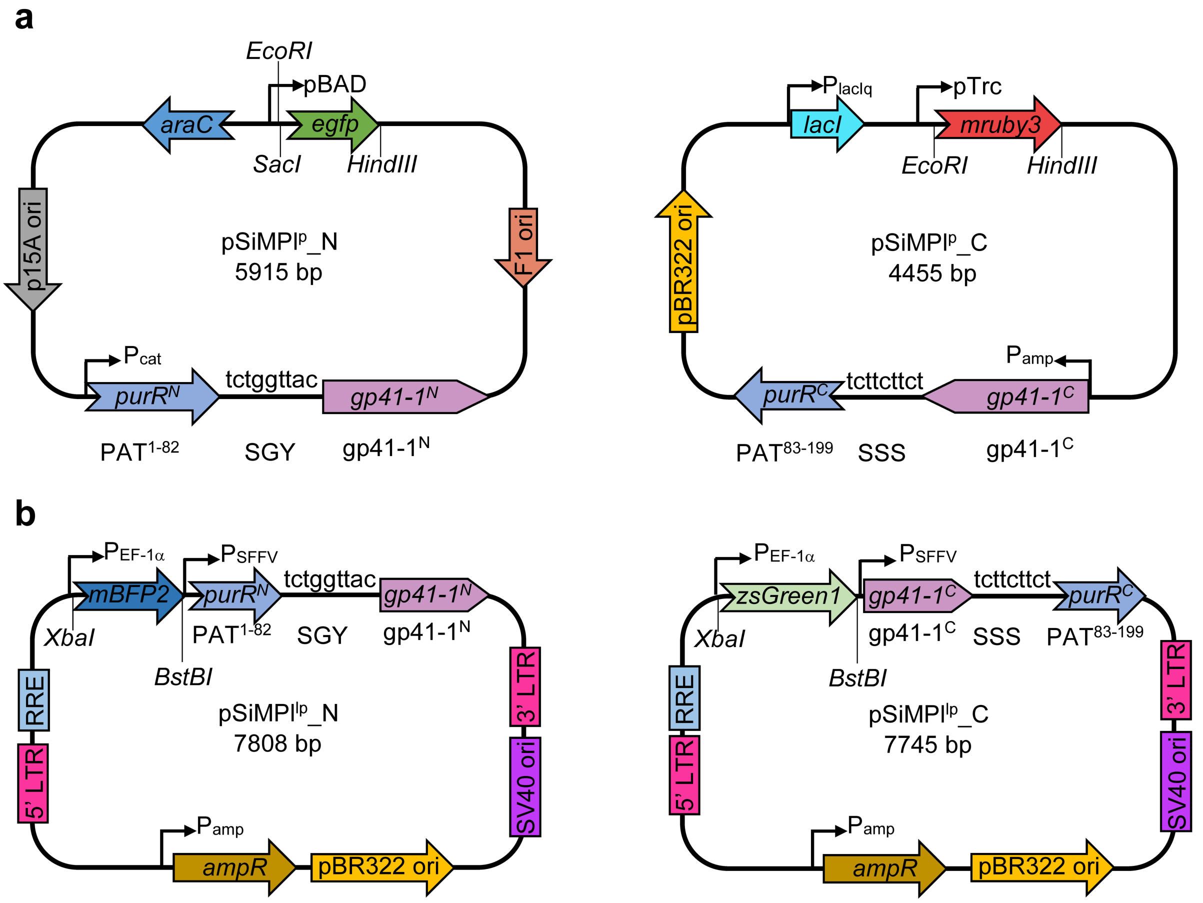

Supplementary Fig. 2 The SiMPl toolkit. **a** Schematic showing the main features found on the SiMPl plasmids for use with puromycin. **b** Schematic showing the main features found on the SiMPl lentiviral vectors for use with puromycin in mammalian cells.

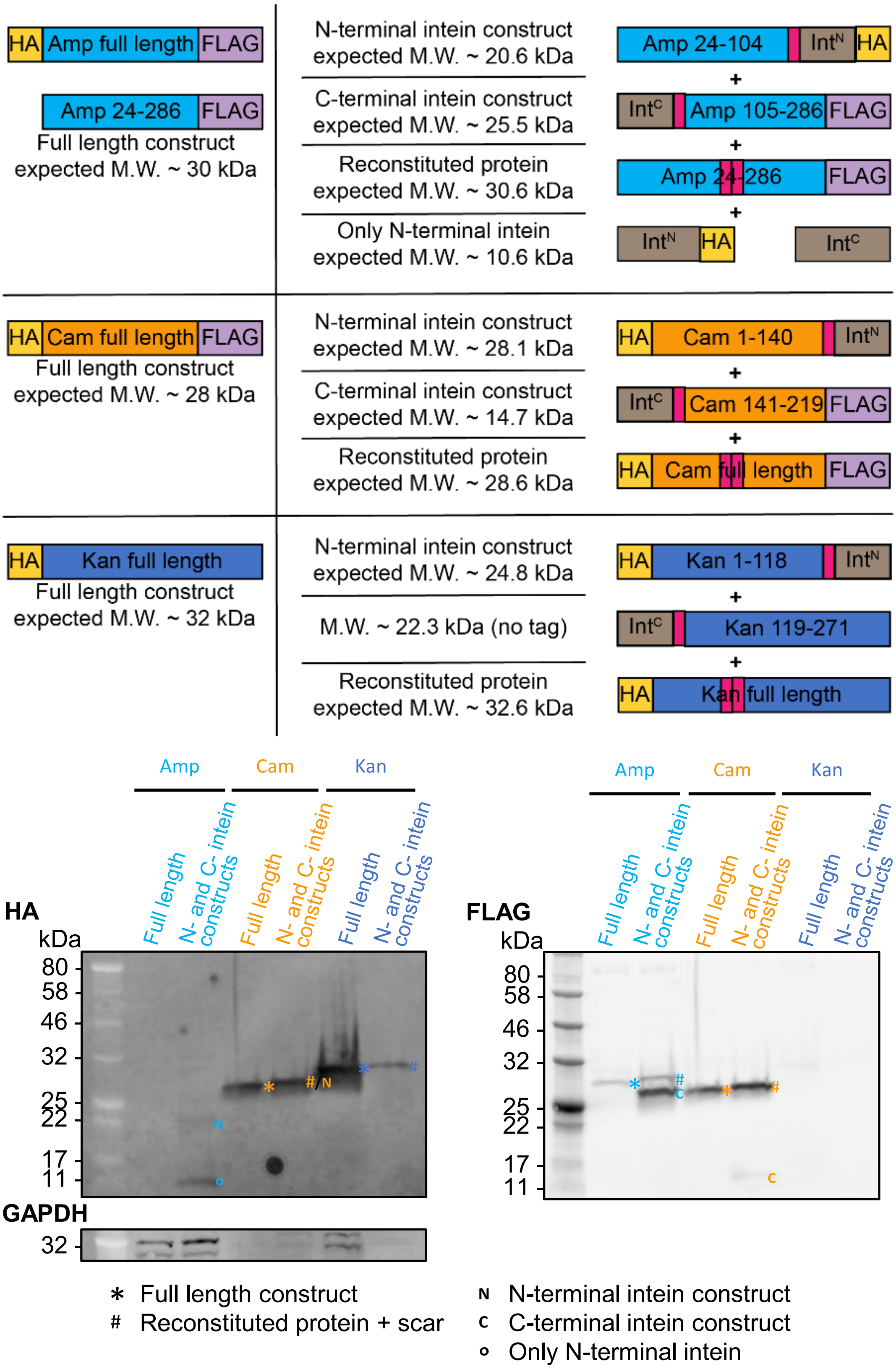

Supplementary Fig. 3 Enzyme reconstitution assessed by Western blotting. Upper panel, schematics of all constructs with predicted molecular weights. Lower panel, representative images of the membranes exposed to the indicated antibodies visualized in the Typhoon laser scanner. Source data are provided as a Source Data file.

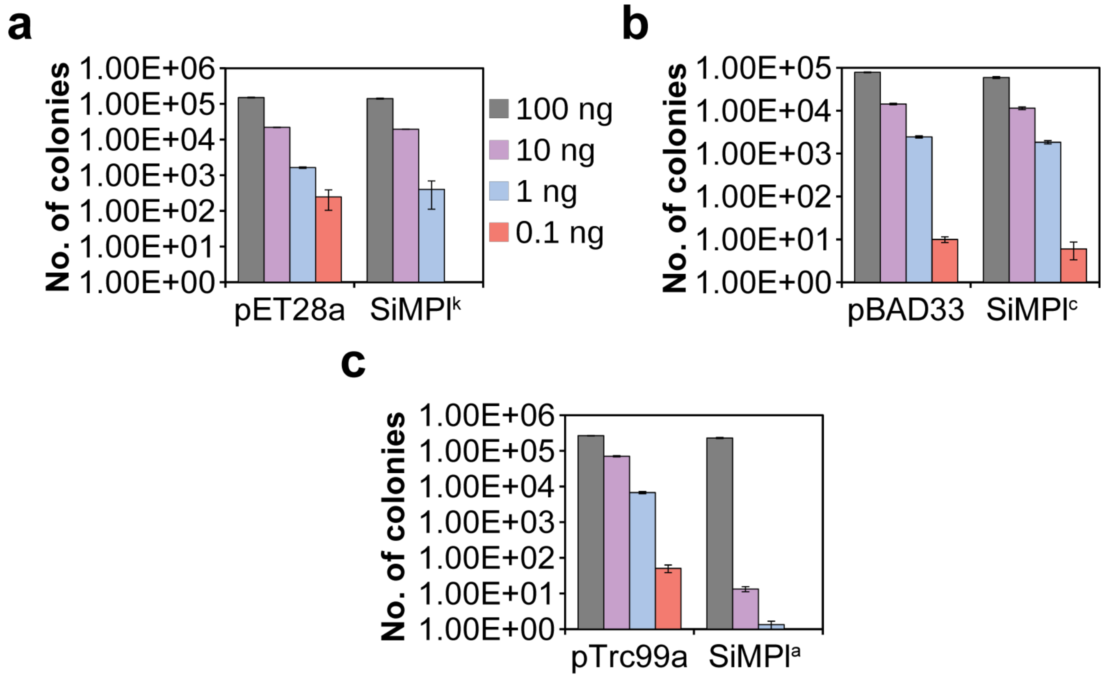

Supplementary Fig. 4 Varying the amount of DNA in the transformation affects the number of colonies obtained with the SiMPl plasmids. **a-c** Bar graph showing the number of colonies obtained after transformation of *E. coli* TOP10 cells with the indicated plasmid DNA at the indicated amounts. Bars represent means, error bars represent standard errors of the mean of three independent experiments. **b-c** Colour code as in (**a**). Source data are provided as a Source Data file.

>sp|P62593|BLAT_ECOLX Beta-lactamase TEM OS=Escherichia coli
MSIQHFRVAL IPFFAAFCLP VFAHPETLVK VKDAEDQLGA RVGYIELDLN SGKILESFRP
EERFPMMSTF KVLLCGAVLS RVDAGQEQLG RRIHYSQNDL VEYSPVTEKH LTDGMTVREL
CSAAITMSDN TAANLLLTTI GGPKELTAFL HNMGDHVTRL DRWEPELNEA IPNDERDTTM
PAAMATTLRK LLTGELLTLA SRQQLIDWME ADKVAGPLLR SALPAGWFIA DKSGAGERGS
RGIIAALGPD GKPSRIVVIY TTGSQATMDE RNRQIAEIGA SLIKHW

>sp|P62577|CAT_ECOLX Chloramphenicol acetyltransferase OS=Escherichia coli
MEKKITGYTT VDISQWHRKE HFEAFQSVAQ CTYNQTVQLD ITAFLKTVKK NKHKFYPAFI
HILARLMNAH PEFRMAMKDG ELVIWDSVHP CYTVFHEQTE TFSSLWSEYH DDFRQFLHIY
SQDVACYGEN LAYFPKGFIE NMFFVSANPW VSFTSFDLNV ANMDNFFAPV FTMGKYYTQG
DKVLMPLAIQ VHHAVCDGFH VGRMLNELQQ YCDEWQGGA

>sp|P00557|KHYB_ECOLX Hygromycin-B 4-O-kinase OS=Escherichia coli
MKKPELTATS VEKFLIEKFD SVSDLMQLSE GEESRAFSFD VGGRGYVLRV NSCADGFYKD
RYVYRHFASA ALPIPEVLDI GEFSESLTYC ISRRAQGVTL QDLPETELPA VLQPVAEAMD
AIAAADLSQT SGFGPFGPQG IGQYTTWRDF ICAIADPHVY HWQTVMDDTV SASVAQALDE
LMLWAEDCPE VRHLVHADFG SNNVLTDNGR ITAVIDWSEA MFGDSQYEVA NIFFWRPWLA
CMEQQTRYFE RRHPELAGSP RLRAYMLRIG LDQLYQSLVD GNFDDAAWAQ GRCDAIVRSG
AGTVGRTQIA RRSAAVWTDG CVEVLADSGN RRPSTRPRAK E

>sp|P13249|PUAC_STRAD Puromycin N-acetyltransferase OS=Streptomyces alboniger
MTEYKPTVRL ATRDDVPRAV RTLAAAFADY PATRHTVDPD RHIERVTELQ ELFLTRVGLD
IGKVWVADDG AAVAVWTTPE SVEAGAVFAE IGPRMAELSG SRLAAQQQME GLLAPHRPKE
PAWFLATVGV SPDHQGKGLG SAVVLPGVEA AERAGVPAFL ETSAPRNLPF YERLGFTVTA
DVEVPEGPRT WCMTRKPGA

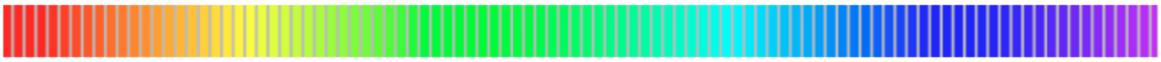

more conserved — less conserved

Supplementary Fig. 5 Evolutionary trace analysis. Amino acid sequence of the indicated enzymes, where each amino acid is coloured according to evolutionary conservation from more (red) to less (blue) conserved. Successful splice sites are highlighted in grey, while unsuccessful ones in orange.

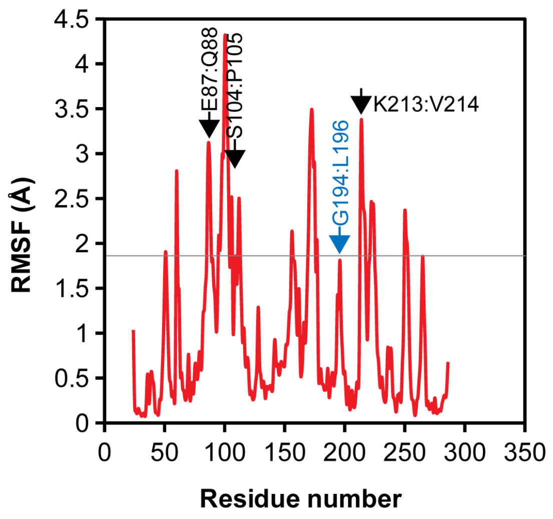

Supplementary Fig. 6 Previously established sites for splitting TEM-1 β-lactamase are not among the best candidates according to the flexibility analysis. Root mean square fluctuation (RMSF) of Cα atoms in TEM-1 β-lactamase (PDB ID: 1zg4) obtained from protein structure fluctuation simulations via the CABS-Flex 2.0 web-server[1]. Flexible regions, within which splice sites were selected, are indicated by black arrows. Previously used residue for splitting the enzyme is indicated by the blue arrow.

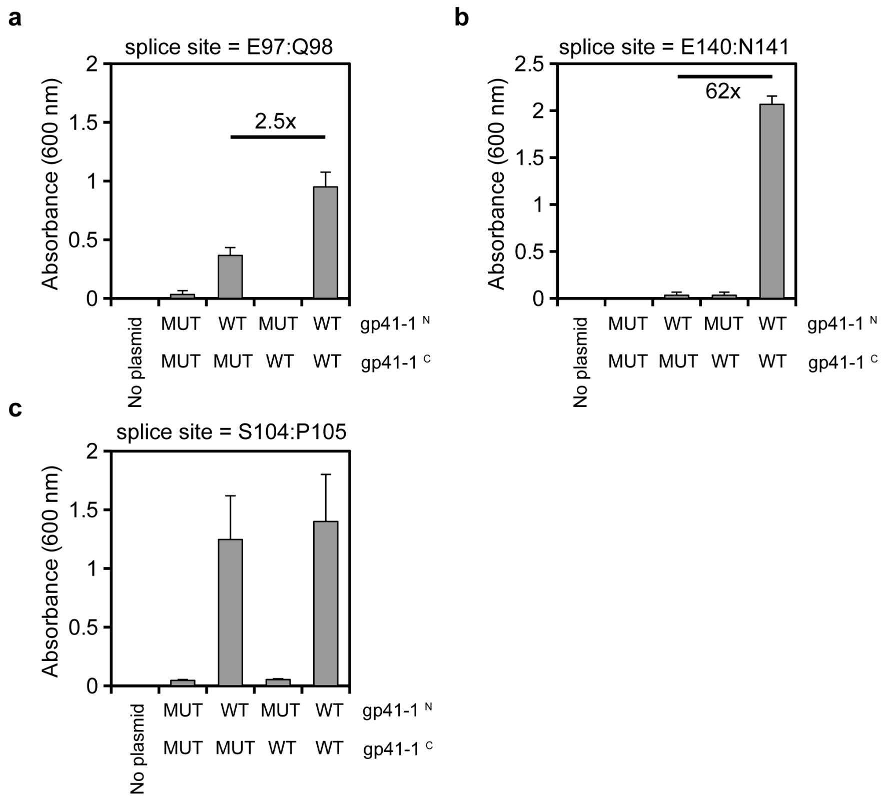

Supplementary Fig. 7 Protein *trans*-splicing is not always needed for enzyme functionality. **a-c** Bar graph showing the values of the absorbance at 600 nm for cultures of *E. coli* TOP10 transformed with the SiMPl plasmids carrying chloramphenicol acetyltransferase (**a**, **b**) and TEM-1 β-lactamase (**c**) split at the indicated sites with either wild type or mutated gp41-1. WT, wild type. gp41-1^{N} MUT, mutation of the conserved cysteine at the very N-terminus of the N-terminal intein fragment to alanine; gp41-1^{C} MUT, mutation of the conserved asparagine at the very C-terminus of the C-terminal intein fragment to alanine. Bars represent means, error bars represent standard errors of the mean of three independent experiments. Source data are provided as a Source Data file.

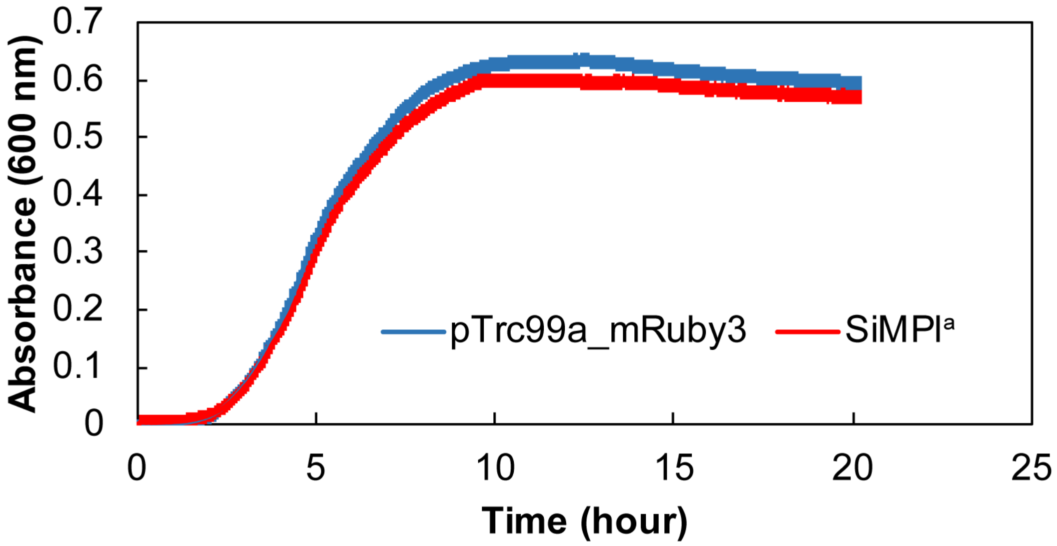

Supplementary Fig. 8 Performance of SiMPl[a] in liquid culture. OD_{600} was measured every 2.4 minutes in a plate reader. Shown are means ± standard error of the mean of three independent measurements. Source data are provided as a Source Data file.

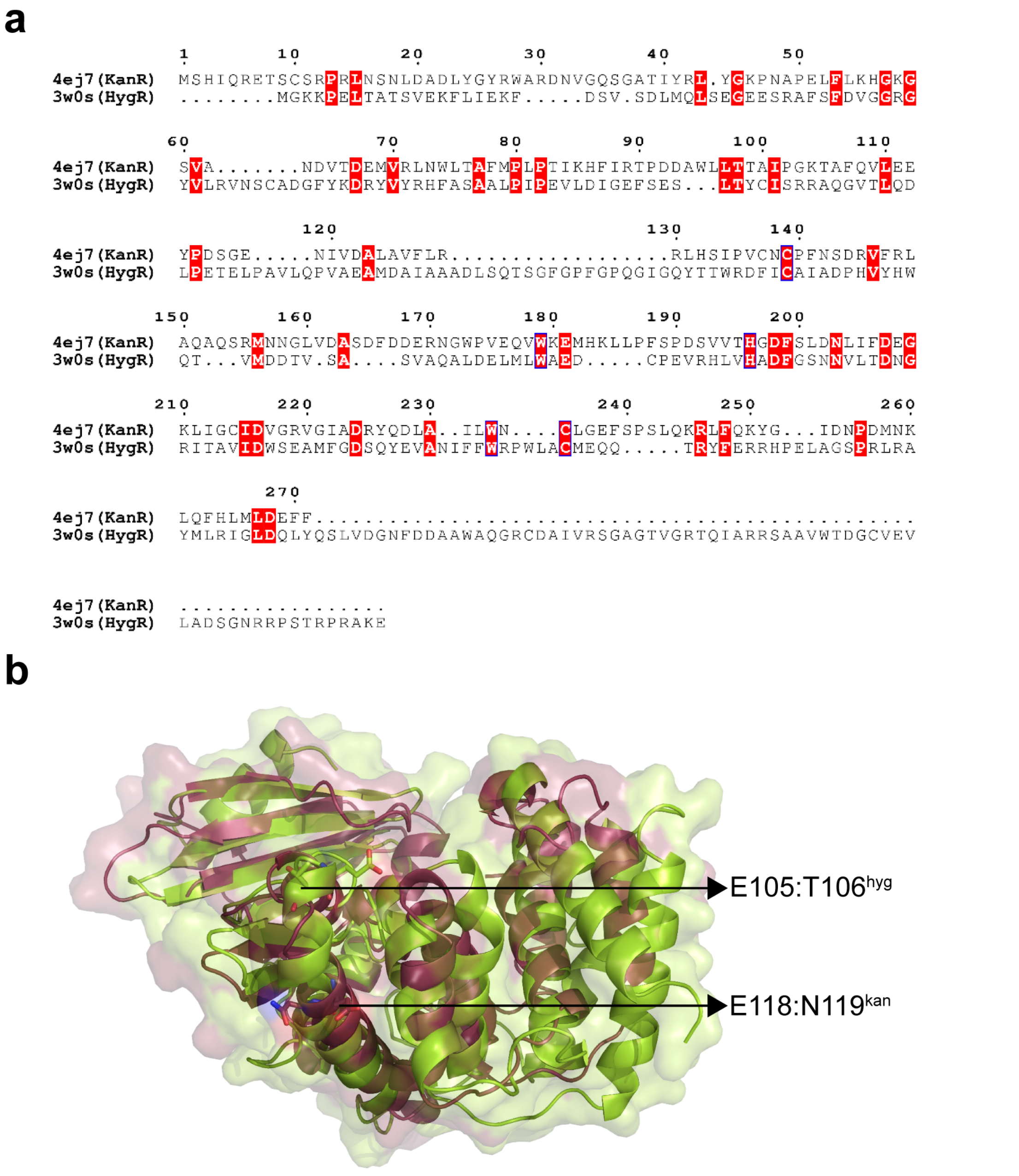

Supplementary Fig. 9 Placing the splice site in hygromycin B phosphotransferase at the same structural position as the splice site in aminoglycoside 3'-phosphotransferase does not lead to a functional reconstituted enzyme. **a** Amino acid sequence alignment of aminoglycoside 3'-phosphotransferase (KanR) and hygromycin B phosphotransferase (HygR) obtained from ESPript 3.0[2]. The numbering, indicating amino acid position, is for aminoglycoside 3'-phosphotransferase. Highlighted in red are identical amino acids. **b** Alignment of the structures of aminoglycoside 3'-phosphotransferase (violet; PDB ID: 4ej7) and hygromycin B phosphotransferase (green; PDB ID: 3w0s). Selected splice sites are highlighted.

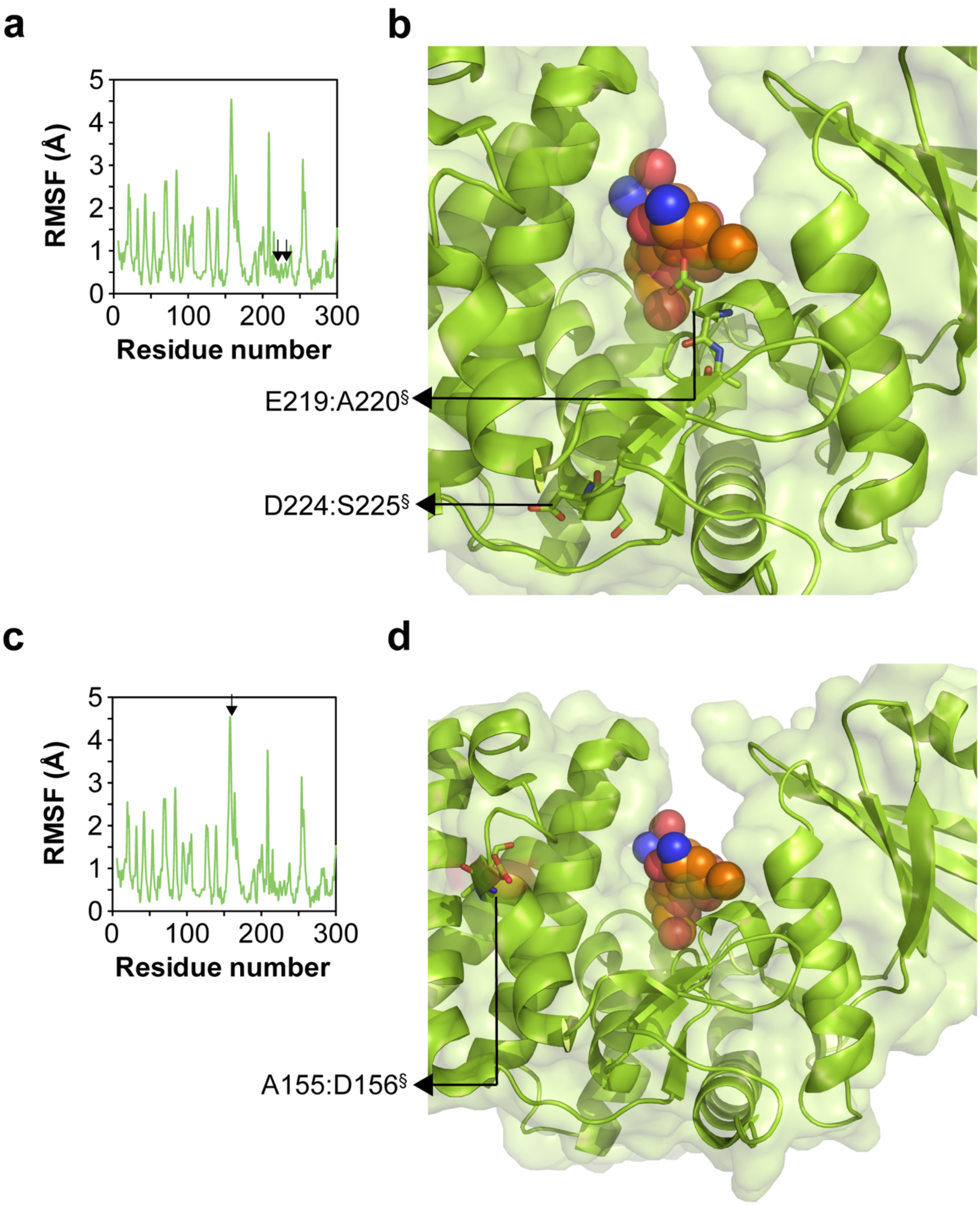

Supplementary Fig. 10 Splice sites selected in regions of low flexibility are not functional. **a** and **c** Root mean square fluctuation (RMSF) of Cα atoms in hygromycin B phosphotransferase (PDB ID: 3w0s) obtained from protein structure fluctuation simulations via CABS-Flex 2.0 web-server[1]. Flexible regions are indicated by black arrows. **b** and **d** Crystal structure of hygromycin B phosphotransferase (PDB ID: 3w0s). Ligand (hygromycin) is represented as coloured spheres. Splice sites are indicated by black arrows. §, splice site that did not support bacterial growth.

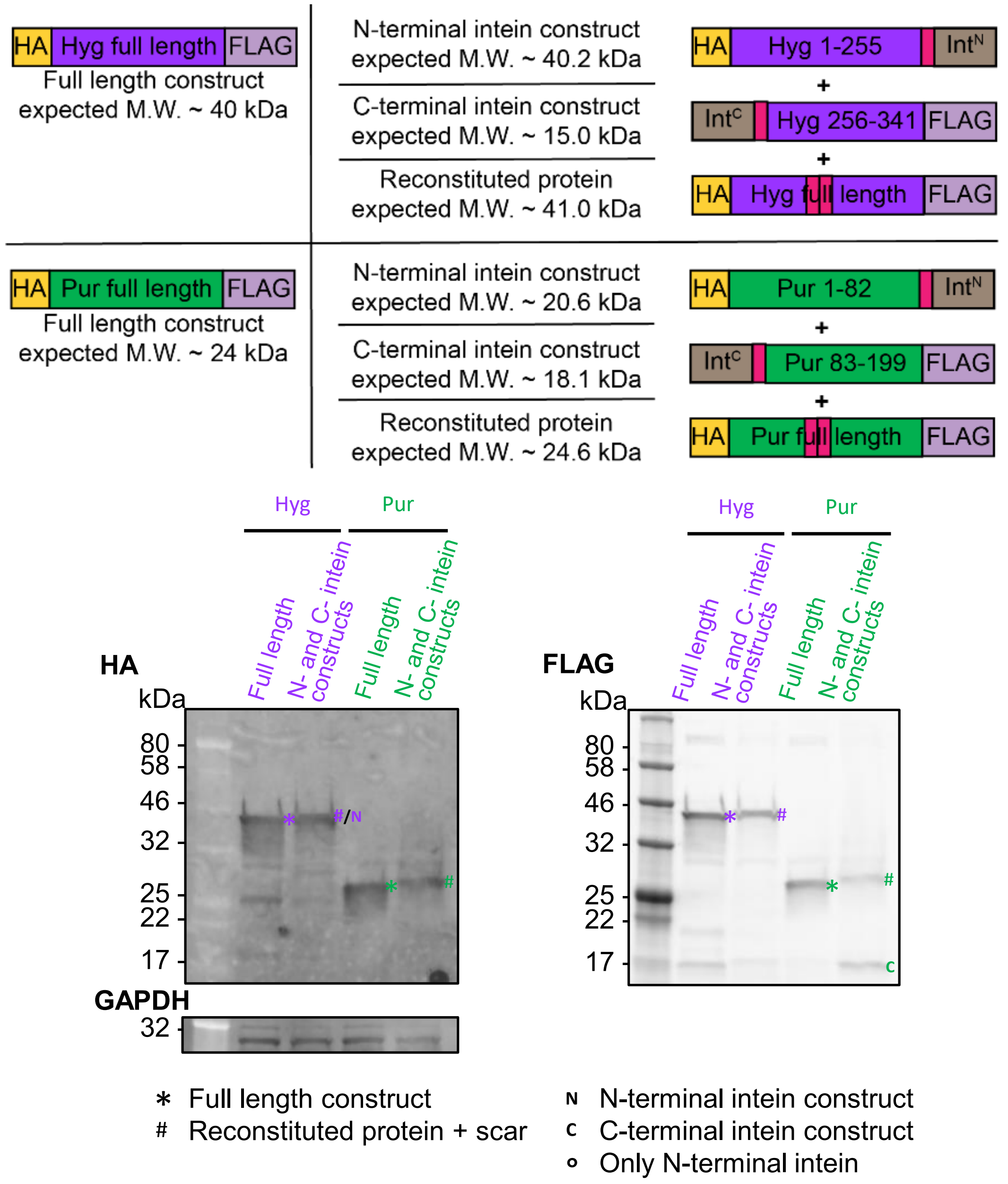

Supplementary Fig. 11 Enzyme reconstitution assessed by Western blotting. Upper panel, schematics of all constructs with predicted molecular weights. Lower panel, representative images of the membranes exposed to the indicated antibodies visualized in the Typhoon laser scanner. Source data are provided as a Source Data file.

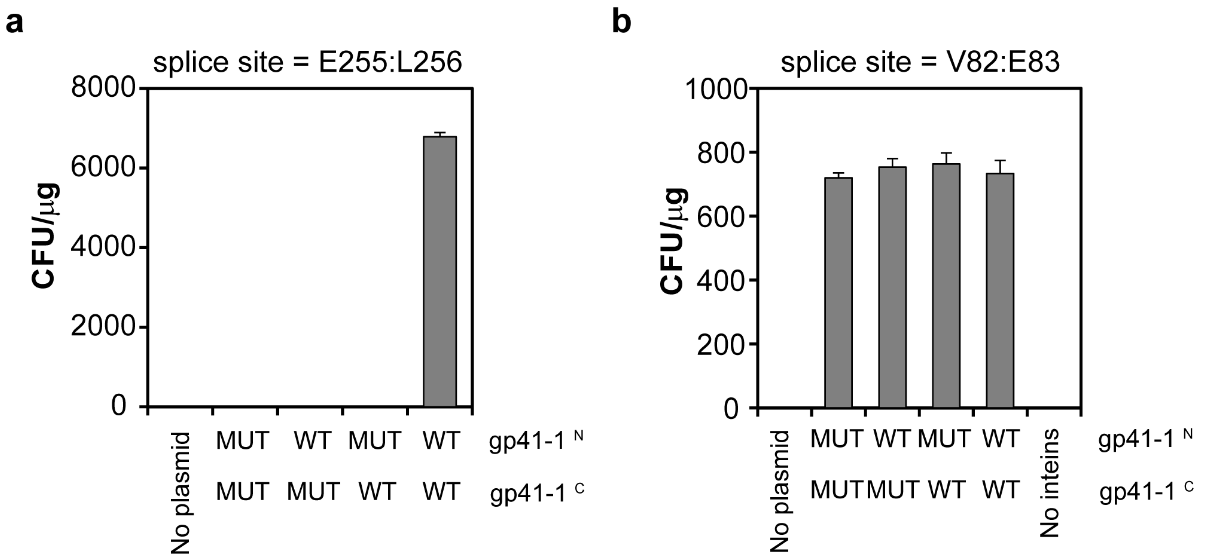

Supplementary Fig. 12 Protein *trans*-splicing is needed for split hygromycin B phosphotransferase but not for puromycin acetyltransferase. **a,b** Bar graph showing the transformation efficiency of the SiMPl plasmids carrying hygromycin B phosphotransferase (**a**) and puromycin acetyltransferase (**b**) split at the indicated position in *E. coli* TOP10 cells with either wild type or mutated gp41-1. WT, wild type. gp41-1^{N} MUT, mutation of the conserved cysteine at the very N-terminus of the N-terminal intein fragment to alanine; gp41-1^{C} MUT, mutation of the conserved asparagine at the very C-terminus of the C-terminal intein fragment to alanine. No inteins, control where the gp41-1 N- and C-terminal fragments were omitted. Bars represent means, error bars represent standard errors of the mean of three independent experiments. Source data are provided as a Source Data file.

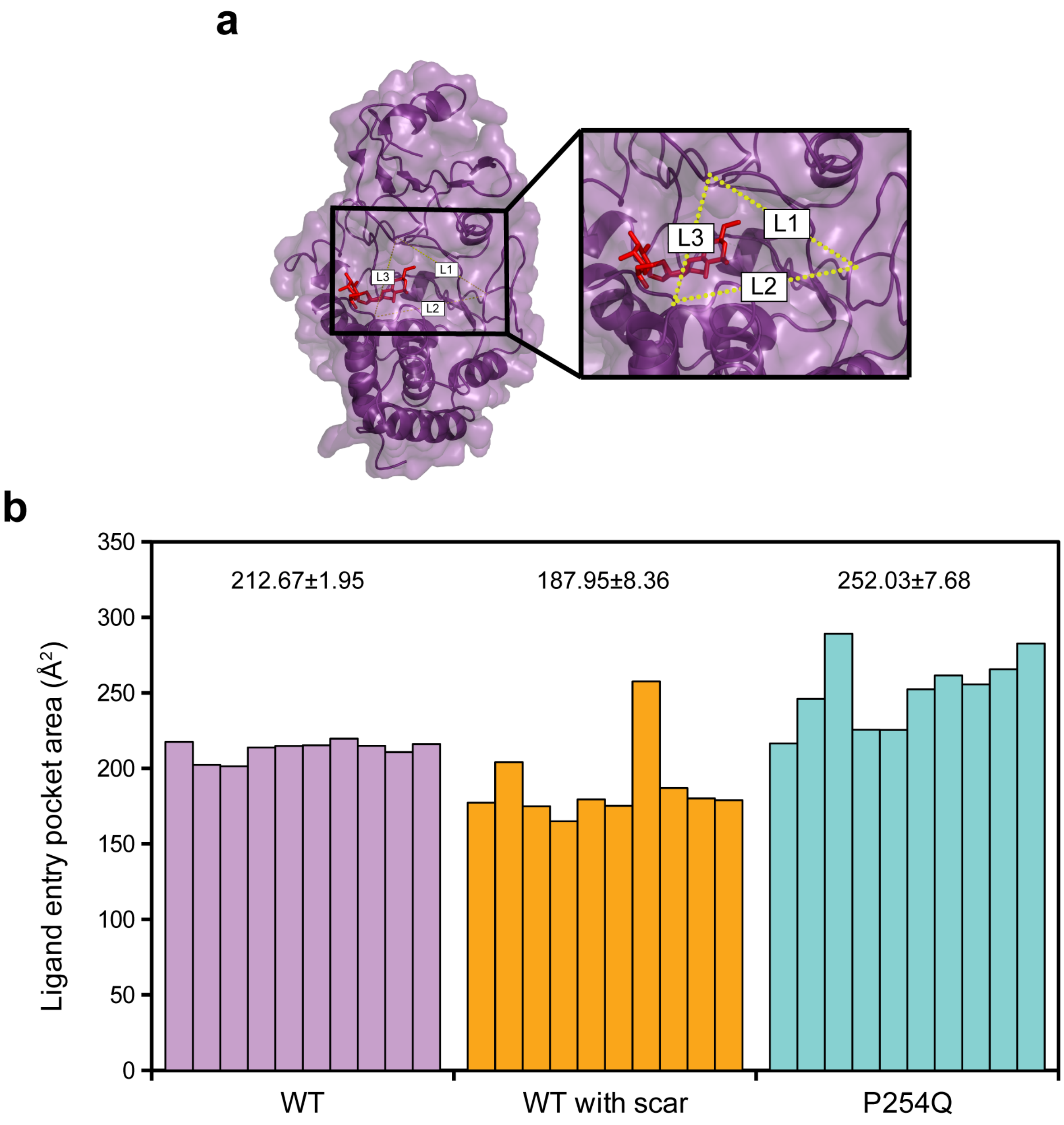

Supplementary Fig. 13 Analysis of the ligand entry pocket in the reconstituted hygromycin B phopshotransferase. **a** Crystal structure of hygromycin B phosphotransferase (PDB ID: 3w0s). Ligand (hygromycin, in red) is shown at the binding pocket. L1, L2 and L3 indicate the edges of a triangle with vertices Gly32, Pro135 and Arg236. This triangle represents the ligand entry pocket. **b** Bar graph showing the ligand entry pocket area for 10 representative structures of the structural ensembles obtained from CABS-flex analysis for the indicated variants of the reconstituted hygromycin B phosphotransferase.

```
Conservation:                   9    999  99  9 9  9  9  999  99                 9 99         9   99 9
tr_Q8NQB1_Q8NQB1_CORGL_Hi    1  MS---PTVLPATQADFPKIVDVLVEAFANDPAFLRWIPQPDPGSAKLRALFELQIEKQYAVAGNIDVARD   67
sp_P13249_PUAC_STRAD_Puro    1  MTEYKPTVRLATRDDVPRAVRTLAAAFADYPATRHTVDPD-RHIERVTELQELFLTRVGLDIGKVWVADD   69
Consensus_aa:                   Mo...PTV..ATpsDhP+hVchLh.AFAs.PAh.+hls.s.....+lp.LbELblp+..hshGpl.VAcD
Consensus_ss:                         eeee  hhhhhhhhhhhhhhh   hhhhhh   h hhhhhhhhhhhhhhhhhhhhh eeeeeee

Conservation:                          99 9  9         9   99     9   9         9   99 9 9 9 99  9
tr_Q8NQB1_Q8NQB1_CORGL_Hi   68  SEGEIVGVALWDRPDGNHSAKDQAAMLPRLVSIFGIKAAQVAWTDLSSARFHPKFPHWYLYTVATSSSAR  137
sp_P13249_PUAC_STRAD_Puro   70  G----AAVAVWTTPESVEAGAVFAEIGPRMAELSGSRLAAQQQMEGLLAPHRPKEPAWFLATVGVSPDHQ  135
Consensus_aa:                   t....htVAlWspP-tsctt.sbA.h.PRhhpl.G.+hA...bh-...A.@+PKbPhW@LhTVthSsshp
Consensus_ss:                       eeeeeee        hhhhhhhhhhhhhhhhhhhhhheeeeeeeee      eeeeeeeee hhh

Conservation:                   9 9 999    9   9        99    9    9 9999           9     9   9
tr_Q8NQB1_Q8NQB1_CORGL_Hi  138  GTGVGSALLNHGIARAGDEAI--YLEATSTRAAQLYNRLGFVPLGYIPSDDDGTPELAMWKPPAMPTV  203
sp_P13249_PUAC_STRAD_Puro  136  GKGLGSAVVLPGVEAAERAGVPAFLETSAPRNLPFYERLGFTVTADVEVPE-GPRTWCMTRKPGA---  199
Consensus_aa:                   GpGlGSAll..Gl..A.c.tl..@LEhotsRsh.hYpRLGFhsht.l.ss-.Gs.phtMh+.Pth...
Consensus_ss:                      hhhhhhhhhhhhhhh   eeeeeee hhhhhhhhh   eeeeeeee     eeeeeee
```

Supplementary Fig. 14 Secondary structure similarity between puromycin and histone acetyltransferases. Amino acid sequence alignment of histone acetyltransferase (tr_Q8NQB1) and puromycin acetyltransferase (sp_P13249) obtained using the PROMALS3D web-server[3]. Numbers above the sequences represent the degree of conservation (from 1, low, to 9, high). Amino acids constituting beta sheets (e) and alpha helices (h) are shown in blue and red, respectively. Consensus_aa, consensus amino acid sequence; Consensus_ss, consensus secondary structure.

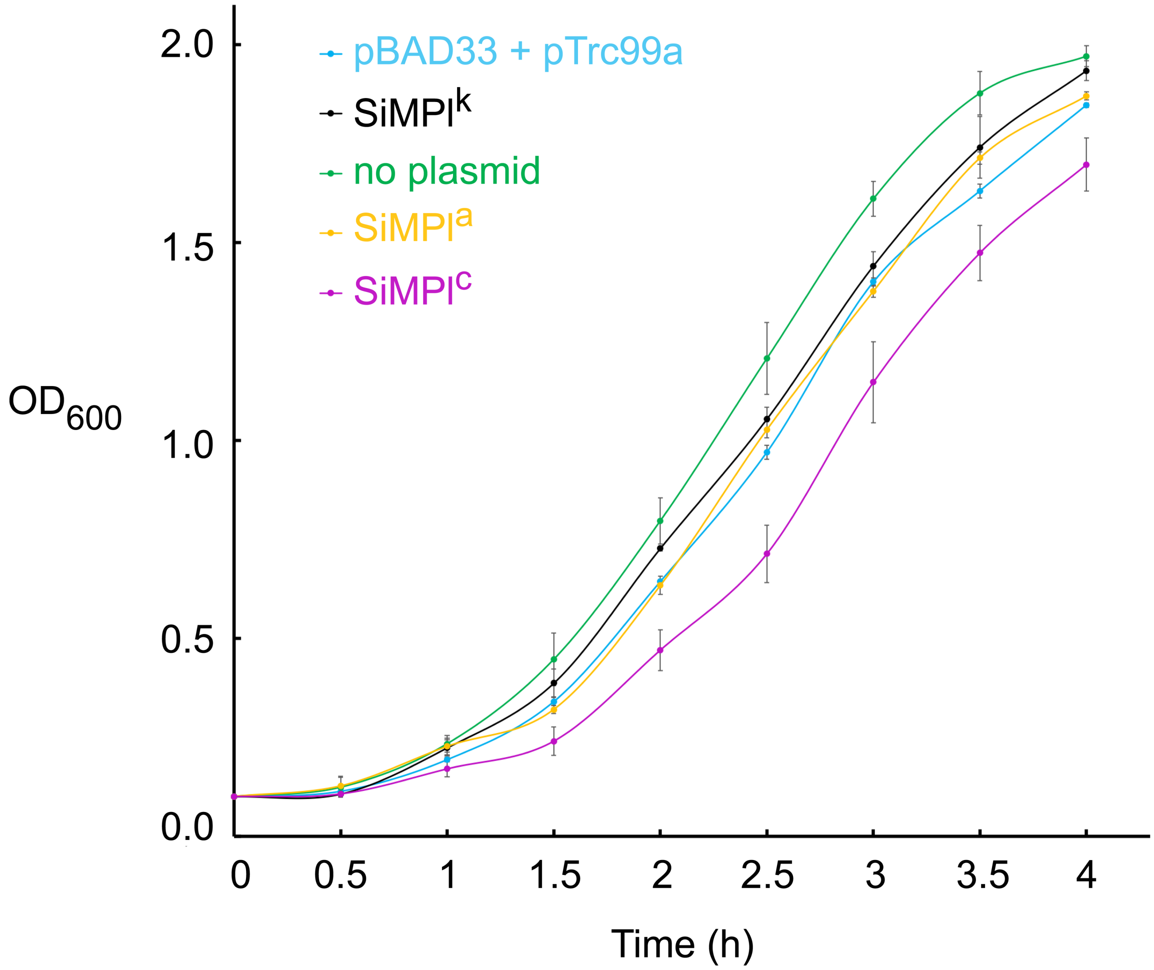

Supplementary Fig. 15 Comparison of the growth of *E. coli* TOP10 cells transformed with either two conventional plasmids, the SiMPl plasmids or no plasmid. Data represent the mean ± standard deviation of three independent experiments. Source data are provided as a Source Data file.

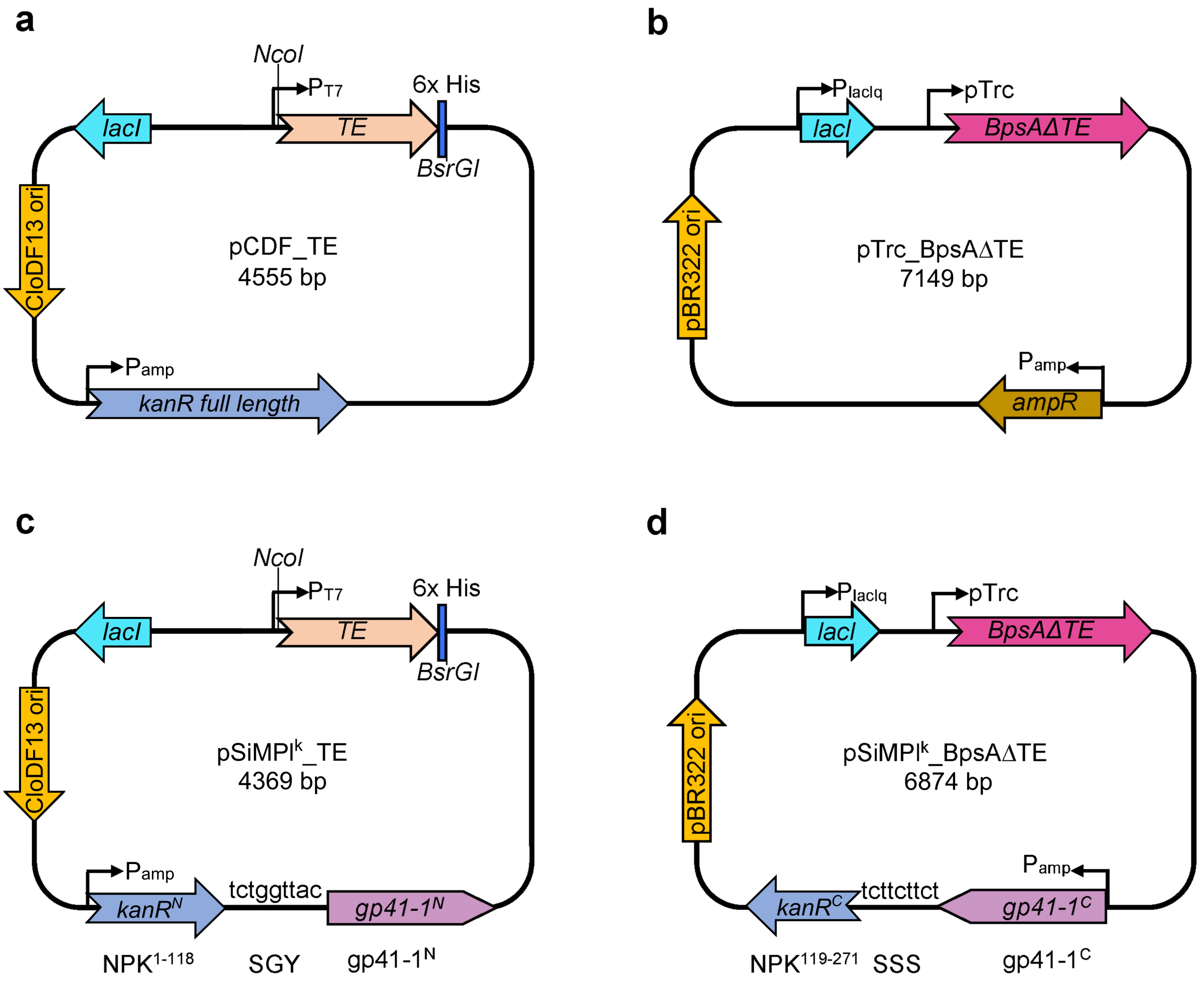

Supplementary Fig. 16 Plasmids used for the production of indigoidine when the truncated synthetase BpsAΔTE and its excised TE domain are expressed from two individual plasmids. **a, b** Traditional plasmids. **c, d** SiMPl plasmids based on kanamycin (SiMPl^{k}).

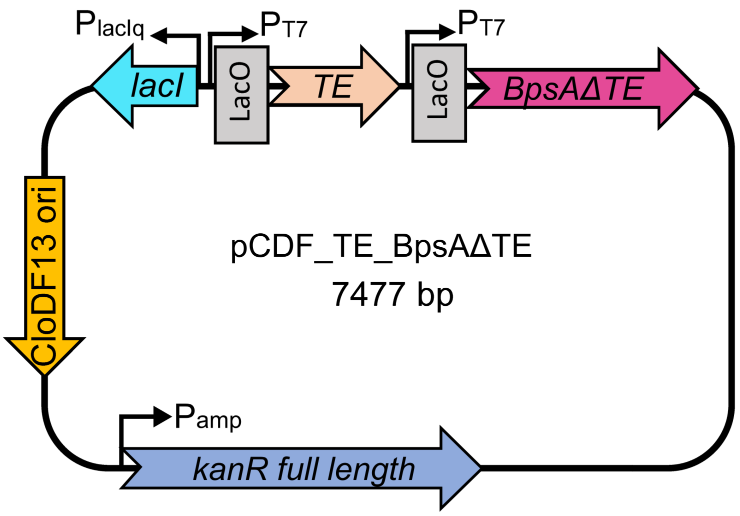

Supplementary Fig. 17 Plasmid used for the production of indigoidine when the truncated synthetase BpsAΔTE and its excised TE domain are expressed from the same plasmid from two independent promoters.

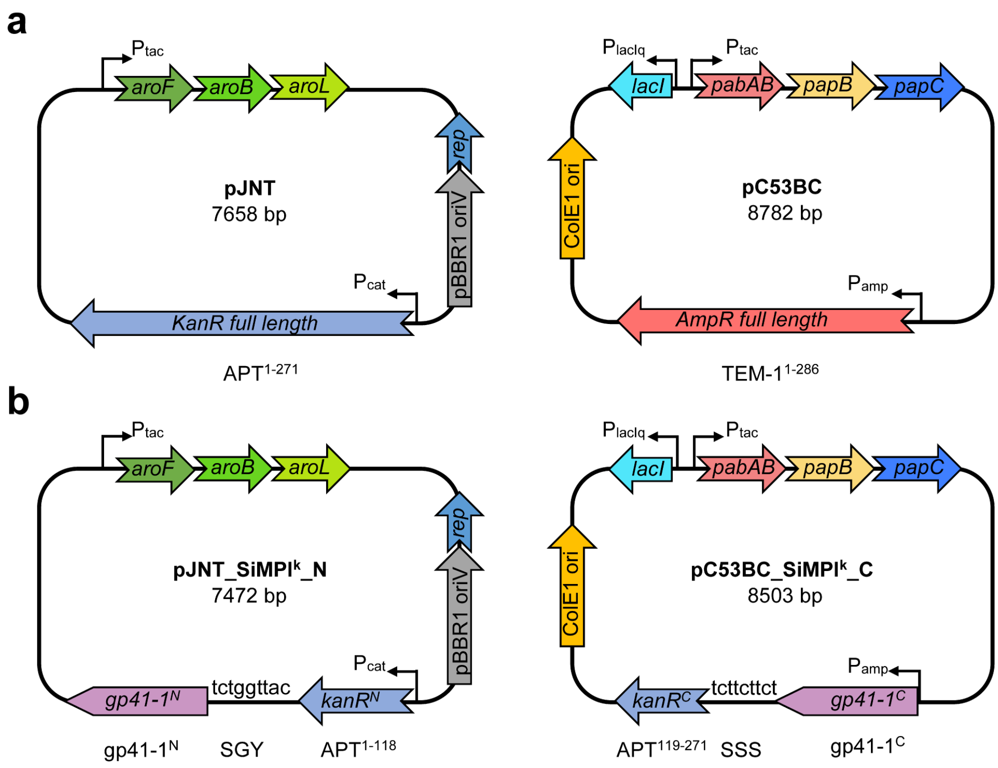

Supplementary Fig. 18 Plasmids used for the production of L-PAPA. **a** Conventional plasmids. **b** SiMPl plasmids based on kanamycin (SiMPl^k).

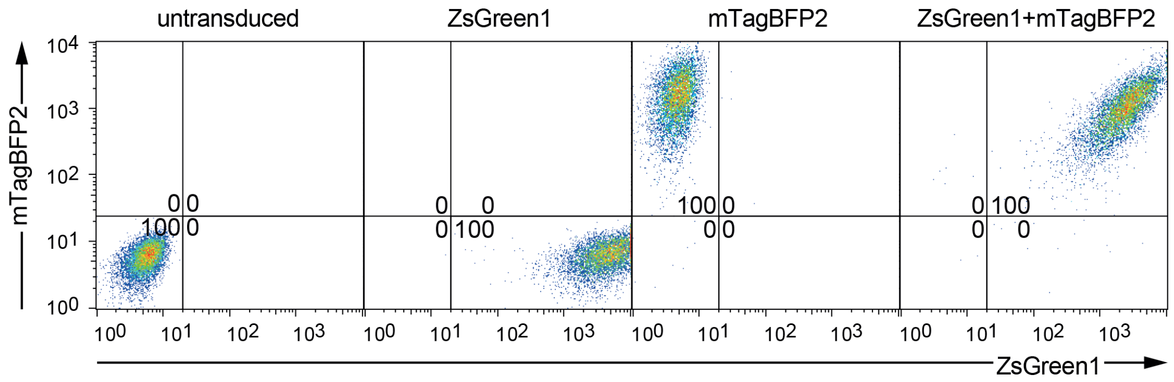

Supplementary Fig. 19 Lentiviruses produced in this study were extremely efficient. TCRα- TCRβ- double negative Jurkat cells were transduced individually or simultaneously with 1 mL lentivirus encoding ZsGreen1 or mTagBFP2 and the puromycin acetyltransferase split at position V82:E83. Flow cytometric analysis of ZsGreen1 and mTagBFP2 expression was performed 4 days after transduction. Gating on living cells was performed using forward versus side scatter. All other virus concentrations gave the same results.

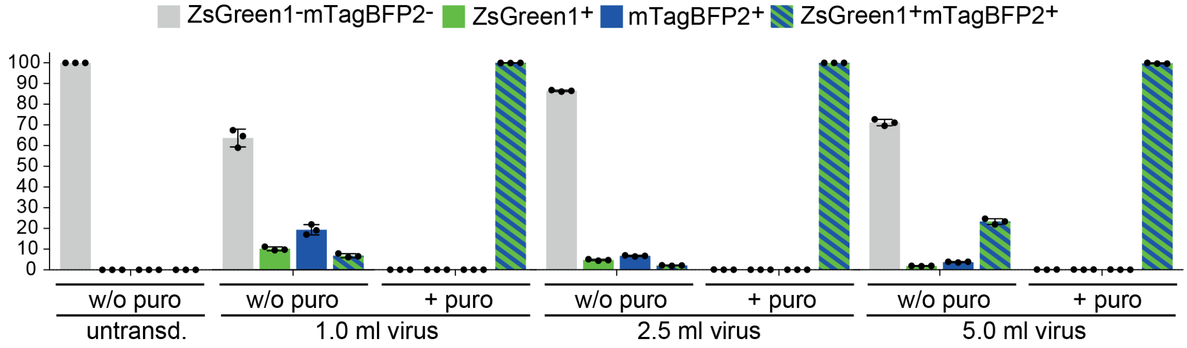

Supplementary Fig. 20 SiMPl can be used to select human T cells with puromycin. Bar graph showing the percentage of cells expressing either one (green or blue), none (grey) or both fluorescent (green with blue stripes) proteins at the indicated conditions for three independent experiments. Values represent mean (± standard deviation). Source data are provided as a Source Data file (Fig.8).

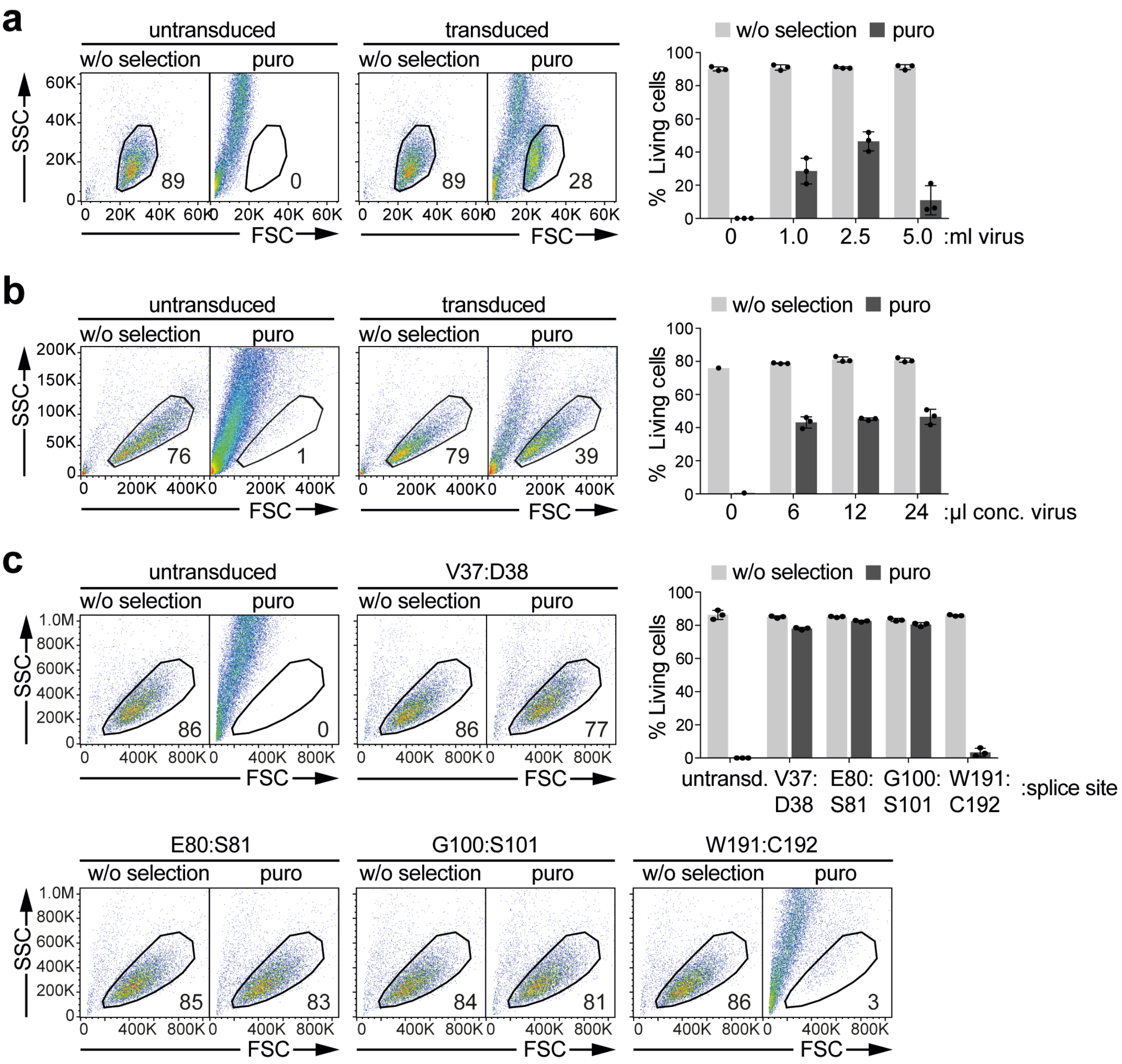

Supplementary Fig. 21 Only cells receiving both halves of the split puromycin acetyltransferase survive selection with puromycin. **a** TCRα$^-$ TCRβ$^-$ double negative Jurkat cells were either left untransduced, or transduced individually or simultaneously with the SiMPl lentiviral vectors encoding mTagBFP2 or ZsGreen1 and the puromycin acetyltransferase split at position V82:E83 and then mixed. The cells were cultured in medium with 0.3 µg/mL puromycin for one day and 0.6 µg/mL puromycin for additional 6 days or kept in medium without puromycin. Flow cytometry analysis of living cells using the CyAn ADP flow cytometer was performed 7 days after removal of puromycin from the culture medium. Left panels, forward versus side scatter (FSC vs SSC). Numbers indicate the percentage of living cells. The bar diagram indicates the percentage of living cells under the indicated conditions for three independent experiments. Error bars represent SD. **b** TCRα$^-$ TCRβ$^-$ double negative Jurkat cells were either transduced individually or simultaneously with the SiMPl lentiviral vectors encoding TCRα or TCRβ and the puromycin acetyltransferase split at position V82:E83 and then mixed. The cells were cultured in the presence or absence of 0.5 µg/mL puromycin for 4 days. Flow cytometry analysis using the Gallios flow cytometer was performed one day after removal of puromycin from the culture medium. Left panels, analysis of living cells using FSC vs SSC gating as in (**a**). The percentage of living cells is indicated. The bar diagram shows the percentage of living cells under the indicated conditions for three independent experiments. Error bars represent SD. **c** TCRα- TCRβ- double negative Jurkat cells were either transduced individually or simultaneously with 2.5 mL lentivirus from the SiMPl vectors encoding TCRα or TCRβ and the differently split puromycin

acetyltransferases as indicated and then mixed. The cells were cultured in medium containing 0.6 µg/mL puromycin or no antibiotic for 7 days. Flow cytometric analysis of living cells using the Gallios flow cytometer was performed 6 days upon removal of puromycin from the culture medium. Left panels show the analysis of living cells using FSC vs SSC gating as in (**a**). The bar diagram shows the percentage of living cells under the indicated conditions for three independent experiments. Error bars represent SD. Source data are provided as a Source Data file.

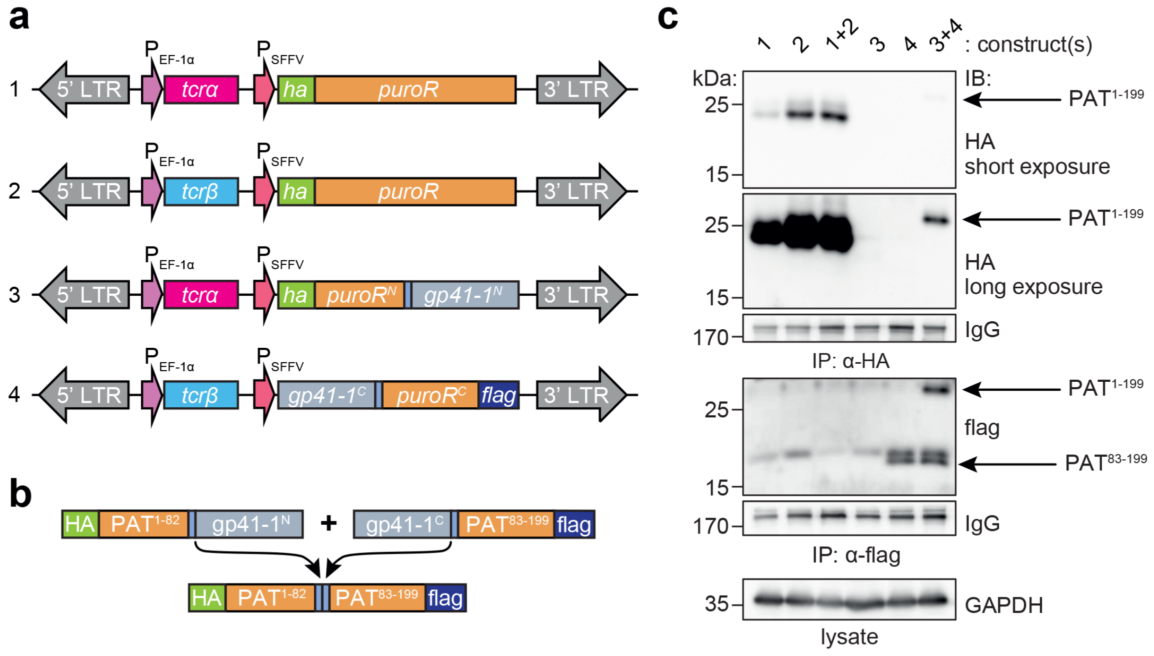

Supplementary Fig. 22 Enzyme reconstitution in Jurkat T cells assessed by Western blotting. **a** Schematics of the constructs. **b** Schematic of enzyme after gp41-1-mediated trans-splicing. **c** Western blot analysis of protein reconstitution. Source data are provided as a Source Data file.

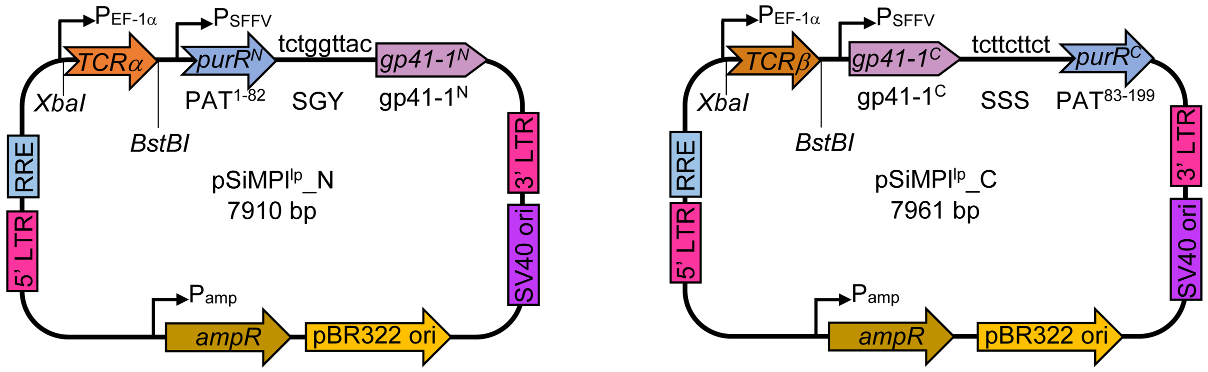

Supplementary Fig. 23 Lentiviral plasmids used to express a TCR of murine origin in Jurkat T cells.

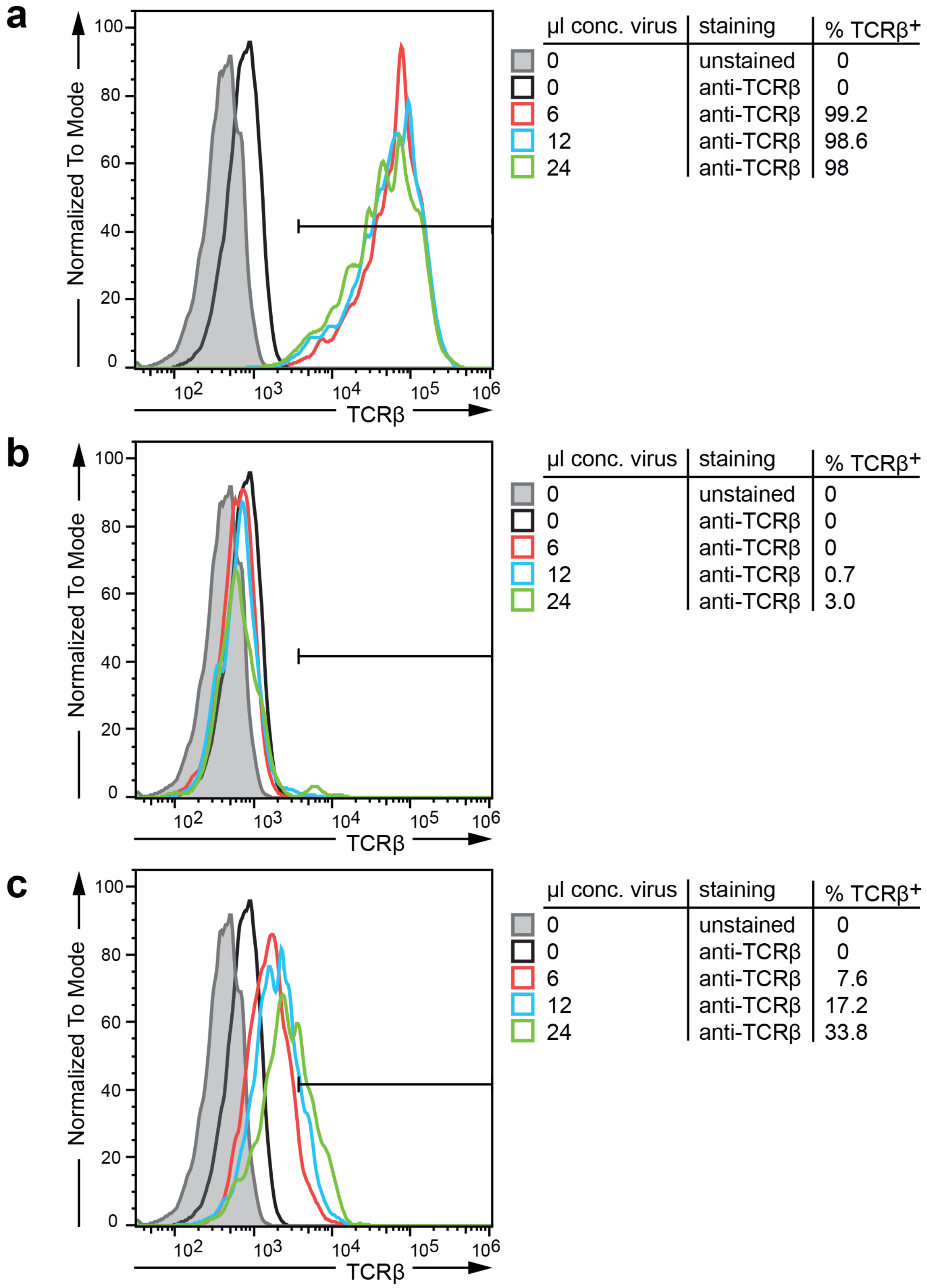

Supplementary Fig. 24 Only cells receiving both TCRα and TCRβ encoded on the SiMPl lentiviral vectors express the complete TCR on the cell surface. **a-c** $TCR\alpha^-$ $TCR\beta^-$ double negative Jurkat cells were transduced with 6, 12 or 24 µL of concentrated lentiviruses produced from both $pSiMPl^{lp}$ vectors shown in Supplementary Figure 22 (**a**), or with only one virus produced from one of these vectors ($p\_SiMPl^{lp}\_N$ in (**b**) and $p\_SiMPl^{lp}\_C$ in (**c**)). 4 days after transduction cell surface staining with APC-coupled antibodies against murine TCRβ was

performed and cells were analysed by flow cytometry. Gating on living cells was performed using forward versus side scatter. The percentage of TCRβ positive living cells is indicated in the table.

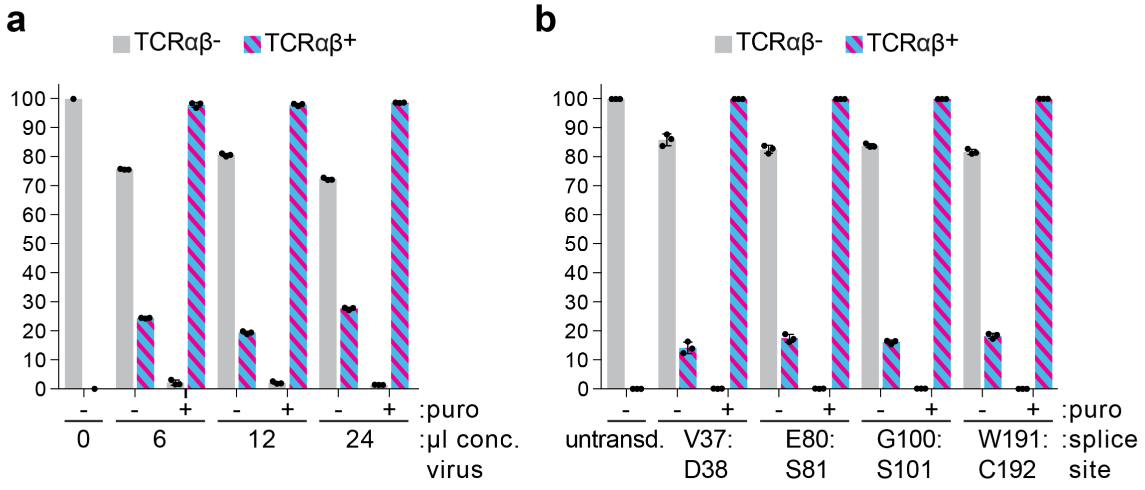

Supplementary Fig. 25 SiMPl allows selecting functional Jurkat T cells expressing a murine TCR on their surface. **a-b** Bar graphs showing the percentage of TCRαβ$^+$ cells for the indicated conditions and the indicated splice sites for three independent experiments. (**a**) Splice site V82:E83. Values represent mean (± standard deviation). Source data are provided as a Source Data file (Fig.9 (for panel a) and Fig.10 (for panel b)).

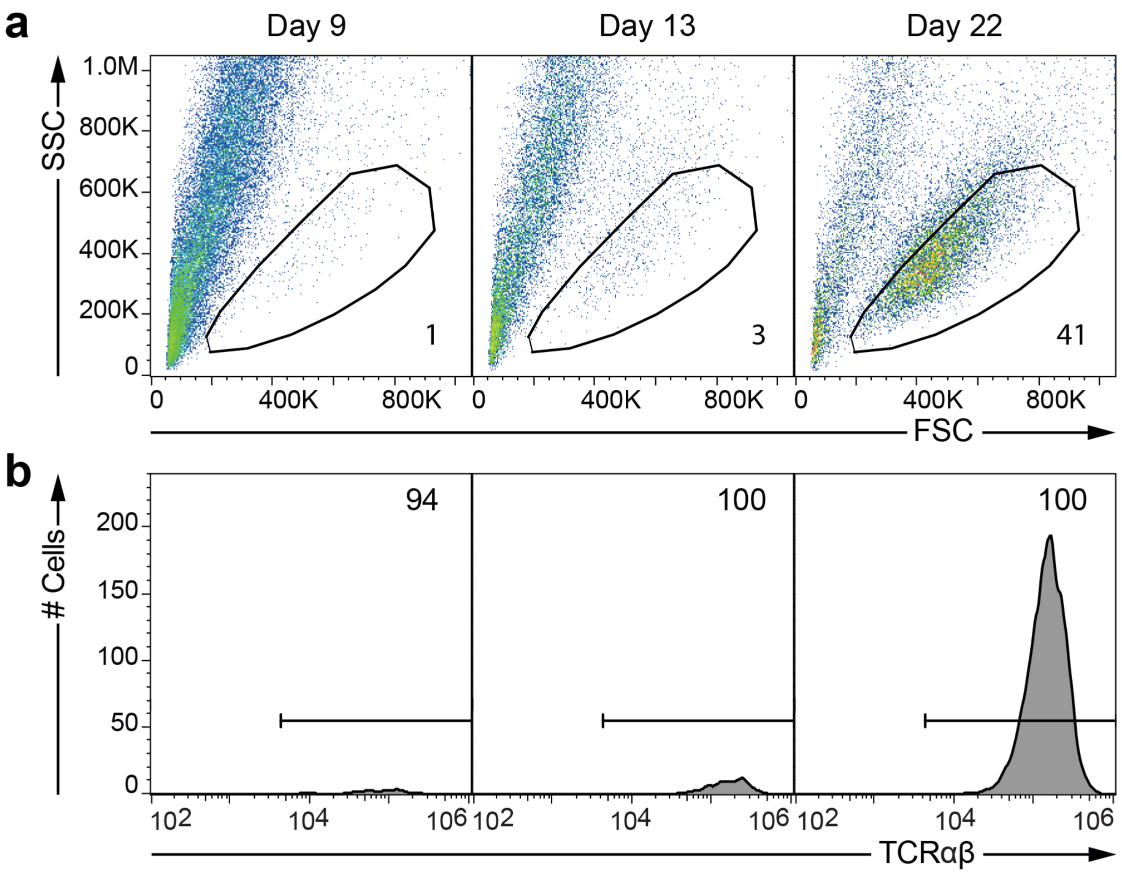

Supplementary Fig. 26 Only few cells expressing puromycin acetyltransferase split at position W191:C192 survive selection with puromycin. **a** TCRα$^-$ TCRβ$^-$ double negative Jurkat cells were either transduced individually or simultaneously with 2.5 mL of lentivirus from the SiMPl vectors encoding TCRα or TCRβ and puromycin acetyltransferase split at position W191:C192 and then mixed. The cells were cultured in medium containing 0.6 µg/mL puromycin for 7 days until puromycin was removed from the culture medium. Cells were stained with APC-coupled anti-TCRβ antibodies at different time points counted from the start of the puromycin selection (day 0) and analysed via flow cytometry. The panels show the analysis of living cells using forward versus side scatter (FSC vs SSC) gating. The numbers indicate the percentage of living cells. **b** Analysis of murine TCRβ expression gated from living cells shown in (**a**). The numbers indicate the percentage of TCR positive cells at the indicated time points.

Supplementary Note 1

With the terminology "conserved amino acid" we do not refer to the conservation of the amino acid as assessed by simple multiple sequence alignment, but rather to a "functionally important amino acid" as assessed by the evolutionary trace method[4], which combines multiple sequence alignment and phylogenetic analyses.

Supplementary Table 1. Primers used in the present study

Primer Name	Sequence (5'-3')	Annealing temperature (ºC)*	Notes
*SacI*_RBS_ATG_EGFP_FP	tttgagctcgctaattgagtaaggccaggatggtgagcaagggcgaggagctgttcaccggggtggtgccc	72	To get eGFP gene with *SacI* and *HindIII* restriction sites.
*HindIII*_TAA_EGFP_RP	tttaagcttttacttgtacagctcgtccatgccgagagtgatcccggcggcgg		
*EcoRI*_mRuby_FP	aaagaattcgtgtctaagggcgaagagctgatcaaggaaaatatgcgtatgaaggtgg	72	To get mRuby gene with *EcoRI* and *HindIII* restriction sites.
*HindIII*_mRuby_RP	tttaagcttttacttgtacagctcgtccatgccaccaccaaggttgctgtatttggc		
No CAM_FP	taatttttttaaggcagttattggtgccc	66.1	To get pBAD33_eGFP without *CmR* gene.
No CAM_RP	tttagcttccttagctcctgaaaatctcg		
No AMP_FP	taactgtcagaccaagtttactcatatatactttag	64.7	To get pTrc99a_mRuby without *AmpR* gene.
No AMP_RP	actcttcctttttcaatattattgaagcatttatc		
KAN_1st_FP	gattttcaggagctaaggaagctaaaatgagccatattcaacgggaaacg	65.4	To get 1st half of *KanR* gene. In KAN_1st_FP, 5' has overlapping sequence with pBAD33 backbone while in KAN_1st_RP, 5'
KAN_1st_RP	cagatccaagcagtaaccagattcacctgaatcaggatattcttctaatacc		

			has overlapping sequence with gp41 N-intein.
GP41_Nintein_FP	tctggttactgcttggatctgaaaaccc	66.2	To get gp41 N-intein. In GP41_Nintein_FP, 5' has overlapping sequence with 1st half of *KanR* fragment while in GP41_Nintein_RP, 5' has overlapping sequence with pBAD33 backbone.
GP41_Nintein_RP	caataactgccttaaaaaaattattctttaacatacagacacataccttctttcag		
GP41_Cintein_FP	caataatattgaaaaaggaagagtatgatgctgaaaaaaatcctgaaaatcgaag	62.5	To get gp41 C-intein. In GP41_Cintein_FP, 5' has overlapping sequence with pTrc99a backbone while in GP41_Cintein_RP, 5' has overlapping sequence with 2nd half of *KanR* gene.
GP41_Cintein_RP	ctgccagcgcatcaacaatattagaagaagagttgtgggtcag		
KAN_2nd_FP	aatattgttgatgcgctggcagtg	62	To get 2nd half of *KanR* gene. In KAN_2nd_FP, 5' has overlapping sequence with gp41 C-intein while
KAN_2nd_RP	gtaaacttggtctgacagttagaaaaactcatcgagcatcaaatg		

			in KAN_2nd_RP, 5’ has overlapping sequence with pTrc99a backbone.
BB1_FP	tttagcttccttagctcctgaaaatctcgataactcaaaaaatacgcccgg	72	To get SiMPl_N backbone without 1st half of *KanR* gene but still retaining the N-intein.
BB1_RP	tctggttactgcttggatctgaaaacccaggttcagaccccg		
BB2_FP	taactgtcagaccaagtttactcatatatactttagattgatttaaaacttcatttttaatttaaaaggatctagg	65	To get SiMPl_C backbone without 2nd half of *KanR* gene but still retaining the C-intein.
BB2_RP	agaagaagagttgtgggtcagaatatcgttagcgtaaaacaggtggttac		
CAM_FP	attttcaggagctaaggaagctaaaatggagaaaaaaatcactggatataccaccg	72	To get 1st half of *CmR* gene (amino acids 1 - 97). In CAM_FP, 5’ has overlapping sequence with pBAD33 backbone while in CAM_97_98_RP, 5’ has overlapping sequence with N-intein.
CAM_97_98_RP	ttttcagatccaagcagtaaccagactcatggaaaacggtgtaacaagggtgaacactatcccatatcaccagctc		
CAM_140_141_RP	ttttcagatccaagcagtaaccagactcaataaaccctttagggaaataggccaggttttcaccgtaacacgcc		To get 1st half of *CmR* gene (amino acids 1 - 140). In

			CAM_FP, 5’ has overlapping sequence with pBAD33 backbone while in CAM_140_141_RP, 5’ has overlapping sequence with N-intein.
CAM_97_98_FP	tattctgacccacaactcttcttctcaaactgaaacgttttcatcgctctggagtgaataccacgacg	72	To get 2nd half of *CmR* gene (amino acids 98 - 219). In CAM_97_98_FP, 5’ has overlapping sequence with C-intein while in CAM_RP, 5’ has overlapping sequence with pTrc99 backbone.
CAM_140_141_FP	tattctgacccacaactcttcttctaatatgtttttcgtctcagccaatccctgggtgagtttcaccag		To get 2nd half of *CmR* gene (amino acids 141 - 219). In CAM_140_141_FP, 5’ has overlapping sequence with C-intein while in CAM_RP, 5’ has
CAM_RP	tatatgagtaaacttggtctgacagttacgccccgccctgccactcatcgcagtactg		

			overlapping sequence with pTrc99 backbone.
AMP_FP	attttcaggagctaaggaagctaaaatgagtattcaacatttccgtgtcgcccttattcccttttttgcggc	72	To get 1st half of *AmpR* gene (amino acids 1 - 87). In AMP_FP, 5' has overlapping sequence with pBAD33 backbone while in AMP_87_88_RP, 5' has overlapping sequence with N-intein.
AMP_87_88_RP	ttttcagatccaagcagtaaccagactcttgcccggcgtcaacacgggataataccgcgc		
AMP_104_105_RP	ttttcagatccaagcagtaaccagatgagtactcaaccaagtcattctgagaatagtgtatgcggcgaccgag		To get 1st half of *AmpR* gene (amino acids 1 - 104). In AMP_FP, 5' has overlapping sequence with pBAD33 backbone while in AMP_104_105_RP, 5' has overlapping sequence with N-intein.
AMP_213_214_RP	ttttcagatccaagcagtaaccagatttatccgcctccatccagtctattaattgttgccgggaagc		To get 1st half of *AmpR* gene (amino acids 1 - 213). In AMP_FP, 5' has

			overlapping sequence with pBAD33 backbone while in AMP_213_214_RP, 5’ has overlapping sequence with N-intein.
AMP_87_88_FP	tattctgacccacaactcttcttctcaactcggtcgccgcatacactattctcagaatgacttggttgag	58.5	To get 2nd half of *AmpR* gene (amino acids 88 - 286). In AMP_87_88_FP, 5’ has overlapping sequence with C-intein while in AMP_RP, 5’ has overlapping sequence with pTrc99 backbone.
AMP_104_105_FP	tattctgacccacaactcttcttctccagtcacagaaaagcatcttacggatggcatgacagtaagagaattatgc		To get 2nd half of *AmpR* gene (amino acids 105 - 286). In AMP_104_105_FP, 5’ has overlapping sequence with C-intein while in AMP_RP, 5’ has

			overlapping sequence with pTrc99 backbone.
AMP_213_214_FP	tattctgacccacaactcttcttctgttgcaggaccacttctgcgc		To get 2nd half of *AmpR* gene (amino acids 214 - 286). In AMP_213_214_FP, 5' has overlapping sequence with C-intein while in AMP_RP, 5' has overlapping sequence with pTrc99 backbone.
AMP_RP	tatatgagtaaacttggtctgacagttaccaatgcttaatcagtgaggcacc		
HYG_FP	attttcaggagctaaggaagctaaaatgggtaaaaagcctgaactcaccgcgacgtctgtcg	72	To get 1st half of *HygR* gene (amino acids 1 - 105). In HYG_FP, 5' has overlapping sequence with pBAD33 backbone while in HYG_105_106_RP, 5' has overlapping sequence with N-intein.
HYG_RP	atgagtaaacttggtctgacagttattattcctttgccctcggacgagtgctggggcg		To get 2nd half of *HygR* gene (amino acids 106 -
HYG_105_106_FP	tattctgacccacaactcttcttctaccgaactgcccgctgttctgcagcc		

HYG_105_106_RP	ttttcagatccaagcagtaaccagattcaggcaggtcttgcaacgtgacaccctgtgc		341). In HYG_105_106_FP, 5’ has overlapping sequence with C-intein while in HYG_RP, 5’ has overlapping sequence with pTrc99 backbone.
HYG_155_156_FP	tattctgacccacaactcttcttctgatccccatgtgtatcactggcaaactgtgatggacgacaccg		To get 2nd half of *HygR* gene (amino acids 156 - 341). In HYG_155_156_FP, 5’ has overlapping sequence with C-intein while in HYG_RP, 5’ has overlapping sequence with pTrc99 backbone.
HYG_207_208_FP	tattctgacccacaactcttcttctaatggccgcataacagcggtcattgactggagcg		To get 2nd half of *HygR* gene (amino acids 208 - 341). In HYG_207_208_FP, 5’ has overlapping sequence with

			C-intein while in HYG_RP, 5’ has overlapping sequence with pTrc99 backbone.
HYG_219_220_FP	tattctgacccacaactcttcttctgcgatgttcggggattcccaatacgaggtcgccaac		To get 2nd half of *HygR* gene (amino acids 220 - 341). In HYG_219_220_FP, 5’ has overlapping sequence with C-intein while in HYG_RP, 5’ has overlapping sequence with pTrc99 backbone.
HYG_224_225_FP	tattctgacccacaactcttcttcttcccaatacgaggtcgccaacatcttcttctggaggccg		To get 2nd half of *HygR* gene (amino acids 225 - 341). In HYG_224_225_FP, 5’ has overlapping sequence with C-intein while in HYG_RP, 5’ has

			overlapping sequence with pTrc99 backbone.
HYG_255_256_FP	tattctgacccacaactcttcttctcttgcaggatcgccgcggctccggg		To get 2nd half of *HygR* gene (amino acids 256 - 341). In HYG_255_256_FP, 5’ has overlapping sequence with C-intein while in HYG_RP, 5’ has overlapping sequence with pTrc99 backbone.
HYG_155_156_RP	ttttcagatccaagcagtaaccagaagcaatcgcgcatatgaaatcacgccatgtagtgtattgaccg		To get 1st half of *HygR* gene (amino acids 1 - 155). In HYG_FP, 5’ has overlapping sequence with pBAD33 backbone while in HYG_155_156_RP, 5’ has overlapping sequence with N-intein.
HYG_207_208_RP	ttttcagatccaagcagtaaccagagtccgtcaggacattgttggagccgaaatccgcgtgc		To get 1st half of *HygR* gene (amino acids 1 - 207).

			In HYG_FP, 5’ has overlapping sequence with pBAD33 backbone while in HYG_207_208_RP, 5’ has overlapping sequence with N-intein.
HYG_219_220_RP	ttttcagatccaagcagtaaccagactcgctccagtcaatgaccgctgttatgcggcc		To get 1st half of *HygR* gene (amino acids 1 - 219). In HYG_FP, 5’ has overlapping sequence with pBAD33 backbone while in HYG_219_220_RP, 5’ has overlapping sequence with N-intein.
HYG_224_225_RP	ttttcagatccaagcagtaaccagaatccccgaacatcgcctcgctccagtcaatg		To get 1st half of *HygR* gene (amino acids 1 - 224). In HYG_FP, 5’ has overlapping sequence with pBAD33 backbone while in HYG_224_225_RP, 5’

			has overlapping sequence with N-intein.
HYG_255_256_RP	ttttcagatccaagcagtaaccagactccggatgcctccgctcgaagtagcgcg		To get 1st half of *HygR* gene (amino acids 1 - 255). In HYG_FP, 5' has overlapping sequence with pBAD33 backbone while in HYG_255_256_RP, 5' has overlapping sequence with N-intein.
PUR_FP§	attttcaggagctaaggaagctaaaatgaccgagtacaagcccacggtgcgcc	72	To get 1st half of *PurR* gene (amino acids 1 - 82). In PUR_FP, 5' has overlapping sequence with pBAD33 backbone while in PUR_82_83_RP, 5' has overlapping sequence with N-intein.
PUR_82_83_RP§	gaacctgggttttcagatccaagcagacgctctccggcgtggtccagaccg		
PUR_112_113_RP§	gaacctgggttttcagatccaagcagaggccttccatctgttgctgcgcggcc		To get 1st half of *PurR* gene (amino acids 1 - 112). In PUR_FP, 5' has

			overlapping sequence with pBAD33 backbone while in PUR_112_113_RP, 5’ has overlapping sequence with N-intein.
PUR_119_120_RP§	gaacctgggttttcagatccaagcacttgggccggtgcggcgccaggagg		To get 1st half of *PurR* gene (amino acids 1 - 119). In PUR_FP, 5’ has overlapping sequence with pBAD33 backbone while in PUR_119_120_RP, 5’ has overlapping sequence with N-intein.
PUR_136_137_RP§	gaacctgggttttcagatccaagcagccctggtggtcgggcgagacgccg		To get 1st half of *PurR* gene (amino acids 1 - 136). In PUR_FP, 5’ has overlapping sequence with pBAD33 backbone while in PUR_136_137_RP, 5’ has overlapping sequence with N-intein.

PUR_153_154_RP[§]	gaacctgggttttcagatccaagcagcgctcggccgcctccactccgggg		To get 1st half of *PurR* gene (amino acids 1 - 153). In PUR_FP, 5’ has overlapping sequence with pBAD33 backbone while in PUR_153_154_RP, 5’ has overlapping sequence with N-intein.
PUR_166_167_RP[§]	gaacctgggttttcagatccaagcagcggggcgcggaggtctccaggaag		To get 1st half of *PurR* gene (amino acids 1 - 166). In PUR_FP, 5’ has overlapping sequence with pBAD33 backbone while in PUR_166_167_RP, 5’ has overlapping sequence with N-intein.
PUR_RP[§]	tatatgagtaaacttggtctgacagtcaggcaccgggcttgcgggtcatgc		To get 2nd half of *PurR* gene (amino acids 83 - 199). In PUR_82_83_FP, 5’ has overlapping sequence with C-intein
PUR_82_83_FP[§]	taacgatattctgacccacaactctgaagcgggggcggtgttcgccgaga		

			while in PUR_RP, 5’ has overlapping sequence with pTrc99 backbone.
PUR_112_113_FP[§]	taacgatattctgacccacaactctctggcgccgcaccggcccaaggagcccg		To get 2nd half of *PurR* gene (amino acids 113 - 199). In PUR_112_113_FP, 5’ has overlapping sequence with C-intein while in PUR_RP, 5’ has overlapping sequence with pTrc99 backbone.
PUR_119_120_FP[§]	taacgatattctgacccacaactctgagcccgcgtggttcctggccaccg		To get 2nd half of *PurR* gene (amino acids 120 - 199). In PUR_119_120_FP, 5’ has overlapping sequence with C-intein while in PUR_RP, 5’ has overlapping sequence with pTrc99 backbone.

PUR_136_137_FP§	taacgatattctgacccacaactctaagggtctgggcagcgccgtcgtgc		To get 2nd half of *PurR* gene (amino acids 137 - 199). In PUR_136_137_FP, 5’ has overlapping sequence with C-intein while in PUR_RP, 5’ has overlapping sequence with pTrc99 backbone.
PUR_153_154_FP§	taacgatattctgacccacaactctgccggggtgcccgccttcctggaga		To get 2nd half of *PurR* gene (amino acids 154 - 199). In PUR_153_154_FP, 5’ has overlapping sequence with C-intein while in PUR_RP, 5’ has overlapping sequence with pTrc99 backbone.
PUR_166_167_FP§	taacgatattctgacccacaactctaacctcccttctacgagcggctcggc		To get 2nd half of *PurR* gene (amino acids 167 - 199). In

			PUR_166_167_FP, 5' has overlapping sequence with C-intein while in PUR_RP, 5' has overlapping sequence with pTrc99 backbone.
HArem_FP	taggcggccgcgtcgacaatcaacctctgg	72	To get the fragment of woodchuck hepatitis virus posttranscriptional regulatory element (WPRE) to core promoter for human elongation factor-1α (EF-1α) when using pOSY082 as template
HArem_RP	catggtggcgaattcgctagctctagactgtgttctggcggc		
SFFVprom_FP	taagaattcgaagccaccagctagctgcagtaacgccattttgc	72	To get the fragment of spleen focus-forming virus long terminal repeat promoter (SFFV) when using pOSY082 as template
SFFVprom_RP	catggtggatccaccggtagatccaccggccg		

TCRα_FP	tagagctagcgaattcgccaccatgaacacttctccagctttagtgactgtgatgctgctgttcatgcttgagagg	72	To get *TCRα* gene. In TCRα_FP, 5’ has overlapping sequence with EF-1α promoter while in TCRα_RP, 5’ has overlapping sequence with SFFV promoter.
TCRα_RP	gctagctggtggcttcgaattcttatcaactggaccacagcctcagcgtcatgagcagg		
TCRβ_FP	tagagctagcgaattcgccaccatgggctccaggctcttcttcgtgctctccagtctcc	72	To get *TCRβ* gene. In TCRβ_FP, 5’ has overlapping sequence with EF-1α promoter while in TCRβ_RP, 5’ has overlapping sequence with SFFV promoter.
TCRβ_RP	gctagctggtggcttcgaattcttaggaattttttttcttgaccatggccatcagcaccaggccactgacc		
mamPURN_FP	tggatctaccggtggatccaccatgatgaccgagtacaagcccacggtgcgcc	72	To get 1st half of *PurR* gene (amino acids 1 - 82) plus N-intein. In mamPURN_FP, 5’ has overlapping sequence with SFFV promoter while in mamPURN_RP, 5’ has
mamPURN_RP	agaggttgattgtcgacgcggccgcctattattctttaacatacagacacataccttctttcagaccaccggag-<br>-atgttcatttcaccagtctgcgtcgg		

			overlapping sequence with WPRE. Template: SiMPl_Pur_NC
mamPURC_FP	tggatctaccggtggatccaccatgatgctgaaaaaaatcctgaaaatcgaagagctggatgaacgtgaactgatcgatattgagg	72	To get C-intein plus 2nd half of *PurR* gene (amino acids 83 - 199). In mamPURC_FP, 5' has overlapping sequence with SFFV promoter while in mamPURC_RP, 5' has overlapping sequence with WPRE. Template: SiMPl_Pur_NC
mamPURC_RP	agaggttgattgtcgacgcggccgcctatcaggcaccgggcttgcgggtcatgcacc		
Ind_BB1_FP	taaaagcttggctgttttggcggatgagagaagattttcagcctg	72	To get SiMPl_Kan_N backbone without *EGFP* gene
Ind_BB1_RP	gagctcgaattcgctagcccaaaaaaacgggtatggagaaacag		
Ind_BB2_FP	taaaagcttggctgttttggcggatgagagaagattttcagcc	72	It's similar to Ind_BB1_FP.
Ind_BB2_RP	catggtctgtttcctgtgtgaaattgttatccgctcacaattccacac		To get SiMPl_Kan_C backbone without *mRuby* gene

Ind_I1_FP	tttttgggctagcgaattcgagctcgtttaactttaataaggagatataccatggagtctagtcgcttcgtccgactgcacgc	72	To get BpsA indigoidine synthetase TE domain. In Ind_I1__FP and Ind_I1_RP, 5’ has overlapping sequences with SiMPl_Kan_N backbone
Ind_I1_RP	atccgccaaaacagccaagcttttactttctgttcgacttaagcattagtggtgatggtgatgatgacctgatccagc		
Ind_I2_FP	aatttcacacaggaaacagaccatgcatcatcaccatcaccacggatcaggtactagtacactgcagg	72	To get rest of BpsA indigoidine synthetase. In Ind_I2__FP and Ind_I2_RP, 5’ has overlapping sequences with SiMPl_Kan_C backbone
Ind_I2_RP	atccgccaaaacagccaagcttttatcactgagccacttctctctccagccgtcggg		
pKAG_BB_RP	CATtttagcttccttagctcctgaaaatctcgataactc	69.8	To get SiMPl_N backbone without 1st half of KanR gene and ‘SGY’ scar but still retaining the N-intein.
pKAG_noSGY_Nint_FP	tgcttggatctgaaaacccaggttcag		
pKBR_noSSS_Cint_RP	agagttgtgggtcagaatatcgttagcgtaaaacaggtggttaccgg	72	Used with BB2_FP to get SiMPl_C backbone without 2nd half of KanR

			gene and 'SSS' scar but still retaining the C-intein.
Pur_37_38_SGY_RP	ttttcagatccaagcagtaaccagaGACGGTGTGGCGCGTGGCGGGGTAG	72	Used with Pur_FP to get 1st half of *PurR* gene (amino acids 1 - 37).
Pur_37_38_SSS_FP	tattctgacccacaactcttcttctGACCCGGACCGCCACATCGAGCGGG	72	Used with Pur_RP to get 2nd half of *PurR* gene (amino acids 38 - 199).
Pur_81_FP	taacgatattctgacccacaactctGTCGAAGCGGGGGCGGTGTTCGCCG	72	Used with Pur_RP to get 2nd half of *PurR* gene (amino acids 81 - 199).
Pur_81_RP	gaacctgggttttcagatccaagcaCTCCGGCGTGGTCCAGACCGCCACC	72	Used with Pur_FP to get 1st half of *PurR* gene (amino acids 1 - 80).
Pur_101_FP	taacgatattctgacccacaactctCGGCTGGCCGCGCAGCAACAGATGG	72	Used with Pur_RP to get 2nd half of *PurR* gene (amino acids 101 - 199).
Pur_101_RP	gaacctgggttttcagatccaagcaACCGCTCAACTCGGCCATGCGCGGG	72	Used with Pur_FP to get 1st half of *PurR* gene (amino acids 1 - 100).

Pur_192_FP	cgctaacgatattctgacccacaacTGCATGACCCGCAAGCCCGGTGCCT	72	Used with Pur_RP to get 2nd half of *PurR* gene (amino acids 192 - 199).
Pur_192_RP	gaacctgggttttcagatccaagcaCCAGGTGCGCGGTCCTTCGGGCACC	72	Used with Pur_FP to get 1st half of *PurR* gene (amino acids 1 - 191).
pSiMPlk-N_fw	GCGAACCGGAATTGC TGATCGGCACGTAAGA	45	To get cat promoter plus the first half of *kanR* gene and the N-intein.
pSiMPlk-N_rev	GAACCCCAGAGTCCCGC TTATTCTTTAACATACA		
pSiMPlk-C_fw	ATGGCCTTTTTGCGTTTCTACAAACTCT	63.7	To get amp promoter plus the C-intein and the second half of *kanR* gene.
pSiMPlk-C_rev	AAGTATATATGAGTAAACTTGGTCTGACAG		
pJNT-amp-fw	TCTTACGTGCCGATCA GCAATTCCGGTTCGC	58.4	To get backbone without the *kanR* gene.
pJNT-amp-rev	TGTATGTTAAAGAATAA GCGGGACTCTGGGGTTC		
pC53BC-amp-fw	ACTCTTCCTTTTTCAATATTATTGA	58.2	To get backbone without the *ampR* gene.
pC53BC-amp-rev	CTGTCAGACCAAGTTTACTCA		
pCDFDuet_MSC2_fwd	taattaacctaggctgctg	57.3	To get pCDFDuet backbone without MCS2.
pCDFDuet_MSC2_rv	catatgtatatctccttcttatacttaac		
pCDFDuet_MSC1_rv	catggtatatctccttattaaagttaaac	59.8	To get pCDFDuet backbone without MCS1.
pCDFDuet_MSC1_fwd	taatgcttaagtcgaacagaaag		
BpsA(pMM64)_pTrc99_fwd	cacacaggaaacagaccatgACTAGTACACTGCAGGAAACAAGC	63.6	

BpsA(pMM64)_(GSG-His6)pTrc99_rv	ggctgaaaatcttctctcagtggtgatggtgatgatgACCTGATCCAGCTTCCCCCAGCAGGTATC		To be used to get *bpsA* with and without the TE domain in pCDFDuet and pTrc99a backbones.
BpsA(E1015)-pCDF1_fwd	ctttaataaggagatataccatgGAGTCTAGTCGCTTCGTCCG		
BpsA(pMM64)_(GSG-His6)pCDF1_rv	ctttctgttcgacttaagcattagtggtgatggtgatgatgACCTGATCCAGCTTCCCCCAGCAGGTATC		
BpsA(Q1014)-pTrc_rv	caggctgaaaatcttctctcaCTGAGCCACTTCTCTCTCCAG		
pCDF_-Spec+Kan_fwd	atatggctcatactcttcctttttcaatattattg	58.4	To get pCDFDuet backbone without the *speR* gene.
pCDF_-Spec+Kan_rv	gagtttttctaatgtctaacaattcgttcaagcc		
Kan_pCDF_fwd	gttagacattagaaaaactcatcgagcatcaaatg	64	To get *kanR* gene.
Kan_pCDF_rv	ggaagagtatgagccatattcaacgggaaac		
pCDF_MSC2-BpsAdTE_fwd	GAAGTGGCTCAGtaattaacctaggctgctgc	58.7	To get pCDFDuet backbone without MCS2.
pCDF_MSC2-BpsAdTE_rv	CTAGTcatatgtatatctccttcttatacttaac		
BpsAdTE_pCDF(MSC2)_fwd	ggagatatacatatgACTAGTACACTGCAGGAAAC	60.1	To get BpsA TE domain.
BpsAdTE_pCDF(MSC2)_rv	cctaggttaattaCTGAGCCACTTCTCTCTCCAG		
SiMPl BFP fw	gcaacgggtttgccgccagaacacagtctagAGAATTCGCCACCATGAGCGAGGAAC	65.7	To get *mTagBFP2* gene. Small letters indicate overhangs.
SiMPl BFP rv	cgttactgcagctagctggtggcttcgaattCTTAGTTCAGCTTGTGGCCCAG		
SiMPl ZsGreen1 fw	gcaacgggtttgccgccagaacacagtctagAGAATTCGCCACCATGGCCCAGTCCAAGCACGG	70.1	To get *zsgreen1* gene. Small letters indicate overhangs.
SiMPl ZsGreen1 rv	cgttactgcagctagctggtggcttcgaattCTCAGGGCAAGGCGGAGCC		

*annealing temperature given for Phusion Flash polymerase, [§]when using these primers, the short linkers between exteins and inteins namely ‘SGY’ and ‘SSS’ were added when amplifying the backbone.

References

1 Kuriata, A. *et al.* CABS-flex 2.0: a web server for fast simulations of flexibility of protein structures. *Nucleic Acids Res* **46**, W338-W343, doi:10.1093/nar/gky356 (2018).

2 Robert, X. & Gouet, P. Deciphering key features in protein structures with the new ENDscript server. *Nucleic Acids Res* **42**, W320-324, doi:10.1093/nar/gku316 (2014).

3 Pei, J., Kim, B. H. & Grishin, N. V. PROMALS3D: a tool for multiple protein sequence and structure alignments. *Nucleic Acids Res* **36**, 2295-2300, doi:10.1093/nar/gkn072 (2008).

4 Wilkins, A., Erdin, S., Lua, R. & Lichtarge, O. Evolutionary Trace for Prediction and Redesign of Protein Functional Sites. *Methods Mol Biol* **819**, 29-42, doi:10.1007/978-1-61779-465-0_3 (2012).
