# Supplementary material for: Split intein-mediated selection of cells containing two plasmids using a single antibiotic
Source: Nat Commun. 2019 Oct 31;10:4967. doi: 10.1038/s41467-019-12911-1 (PMC6823396; doi:10.1038/s41467-019-12911-1)
Supplement: Supplementary file 2 — Reporting Summary [file 41467_2019_12911_MOESM2_ESM.pdf]

nature research

Corresponding author(s): Prof. Barbara Di Ventura

Last updated by author(s): Sep 6, 2019

Reporting Summary

Nature Research wishes to improve the reproducibility of the work that we publish. This form provides structure for consistency and transparency in reporting. For further information on Nature Research policies, see Authors & Referees and the Editorial Policy Checklist .

Statistics

For all statistical analyses, confirm that the following items are present in the figure legend, table legend, main text, or Methods section.

n/a	Confirmed	
☐	☒	The exact sample size (*n*) for each experimental group/condition, given as a discrete number and unit of measurement
☐	☒	A statement on whether measurements were taken from distinct samples or whether the same sample was measured repeatedly
☐	☒	The statistical test(s) used AND whether they are one- or two-sided<br>*Only common tests should be described solely by name; describe more complex techniques in the Methods section.*
☒	☐	A description of all covariates tested
☐	☒	A description of any assumptions or corrections, such as tests of normality and adjustment for multiple comparisons
☐	☒	A full description of the statistical parameters including central tendency (e.g. means) or other basic estimates (e.g. regression coefficient) AND variation (e.g. standard deviation) or associated estimates of uncertainty (e.g. confidence intervals)
☐	☒	For null hypothesis testing, the test statistic (e.g. *F*, *t*, *r*) with confidence intervals, effect sizes, degrees of freedom and *P* value noted<br>*Give P values as exact values whenever suitable.*
☒	☐	For Bayesian analysis, information on the choice of priors and Markov chain Monte Carlo settings
☒	☐	For hierarchical and complex designs, identification of the appropriate level for tests and full reporting of outcomes
☒	☐	Estimates of effect sizes (e.g. Cohen's *d*, Pearson's *r*), indicating how they were calculated

Our web collection on statistics for biologists contains articles on many of the points above.

Software and code

Policy information about availability of computer code

Data collection	Flow cytometry was performed using the Gallios (Beckman Coulter) machine; absorbance measurements were done using the IMPLEN OD600 DiluPhotometer; OD measurements for bacterial growth curve were obtained using the BioTek Synergy H4 Hybrid microplate reader, Indigoidine production was measured using the TECAN microplate reader, Agarose gels and bacterial agar plates were imaged using the analytikjena UVP UVsolo touch; Microscopy images were acquired using the Zeiss AxioObserver wide-field microscope equipped with a cooled CCD-camera 'AxioCam MRm' and alpha-Plan-APOCHROMAT 100x objective; Western Blot images were acquired using Amersham Typhoon biomolecular imager
Data analysis	FlowJo 9.3.2 and 10 (Tree Star) was used to analyze the flow cytometry data; GraphPad Prism 5 (GraphPad Software) was used to make the graphs (lentiviral part only); Microsoft Excel 2016 and LibreOffice Calc 6.2 were used to make the graphs; PyMOL 0. 99, DeLano Scientific LLC, Palo Alto, CA was used to visualize the 3D structure of proteins; CABS-flex 2.0 (http://biocomp.chem.uw.edu.pl/CABSflex2) was used to study the protein Calpha fluctuations; ESPript 3.0 (http://espript.ibcp.fr/ESPript/ESPript/) was used to create multiple sequence alignment figure; SWISS-MODEL (https://swissmodel.expasy.org/) was used to model the 3D structure of hygromycin B phosphotransferase (with scar and mutant); RaptorX web server (http://raptorx.uchicago.edu/) was used to make a homology modelling of 3D structure of puromycin acetyltransferase; Evolutionary Trace Server (http://lichtargelab.org/software/ETserver) was used to perform evolutionary trace analyses; PROMALS3D webserver (http://prodata.swmed.edu/promals3d/promals3d.php) was used to identify structural conservation of proteins. Western Blot images were analysed using ImageQuant.

For manuscripts utilizing custom algorithms or software that are central to the research but not yet described in published literature, software must be made available to editors/reviewers. We strongly encourage code deposition in a community repository (e.g. GitHub). See the Nature Research guidelines for submitting code & software for further information.

Data

Policy information about availability of data

All manuscripts must include a data availability statement. This statement should provide the following information, where applicable:

- Accession codes, unique identifiers, or web links for publicly available datasets
- A list of figures that have associated raw data
- A description of any restrictions on data availability

Plasmids for use in bacteria were deposited at Addgene. Lentiviral vectors can be obtained from the corresponding author.

Field-specific reporting

Please select the one below that is the best fit for your research. If you are not sure, read the appropriate sections before making your selection.

[x] Life sciences [ ] Behavioural & social sciences [ ] Ecological, evolutionary & environmental sciences

For a reference copy of the document with all sections, see nature.com/documents/nr-reporting-summary-flat.pdf

Life sciences study design

All studies must disclose on these points even when the disclosure is negative.

Sample size	Sample sizes were chosen on the basis of an initial pilot experiment and further based on similar experiments previously reported in other publications
Data exclusions	No data were excluded from the analyses
Replication	The number of replicates performed is indicated in each figure legend, where applicable
Randomization	All the samples and the controls were treated using identical protocols side by side. No randomization was used.
Blinding	No blinding was used

Reporting for specific materials, systems and methods

We require information from authors about some types of materials, experimental systems and methods used in many studies. Here, indicate whether each material, system or method listed is relevant to your study. If you are not sure if a list item applies to your research, read the appropriate section before selecting a response.

Materials & experimental systems

n/a	Involved in the study	
[ ]	[x]	Antibodies
[ ]	[x]	Eukaryotic cell lines
[x]	[ ]	Palaeontology
[x]	[ ]	Animals and other organisms
[x]	[ ]	Human research participants
[x]	[ ]	Clinical data

Methods

n/a	Involved in the study	
[x]	[ ]	ChIP-seq
[ ]	[x]	Flow cytometry
[x]	[ ]	MRI-based neuroimaging

Antibodies

Antibodies used	anti-murine TCRβ-allophycocyanin (eBiosciences, catalog number:17-5961-82, clone: H57-597, lot number: E023337); rat anti-HA (1:2000, Cat#11867423001, Sigma); rabbit anti-FLAG (1:2000, Cat# AHP1074, Bio-Rad); mouse anti-GAPDH (1:1000, Cat# G13-61M, SignalChem); AlexaFluor 488-conjugated anti-rat (1:2000, Cat# A-11006, Invitrogen); Cyanine5-conjugated anti-rabbit (1:2000, Cat# A10523, Invitrogen); AlexaFluor 790-conjugated anti-mouse (1:2000, Cat# A28182, Invitrogen)
Validation	All the antibodies were commercially available and were validated by the manufacturer, previous publications and in the present study

Eukaryotic cell lines

Policy information about cell lines

Cell line source(s)	The cell line was a kind gift of Balbino Alarcón, Madrid.

Authentication | Transduction of TCRα and TCRβ induced expression of the T cell receptor on the cell surface of the used cell line. This indicates that the cell line used expresses the components of the CD3 complex and thus is a T cell line.

Mycoplasma contamination | The cell line was not tested for Mycoplasma.

Commonly misidentified lines (See ICLAC register) | *Name any commonly misidentified cell lines used in the study and provide a rationale for their use.*

Flow Cytometry

Plots

Confirm that:

☒ The axis labels state the marker and fluorochrome used (e.g. CD4-FITC).

☒ The axis scales are clearly visible. Include numbers along axes only for bottom left plot of group (a 'group' is an analysis of identical markers).

☒ All plots are contour plots with outliers or pseudocolor plots.

☒ A numerical value for number of cells or percentage (with statistics) is provided.

Methodology

Sample preparation | Cells were cultured in RPMI 1640 GlutaMAX (Thermo Fisher Scientific) containing 10% FCS, HEPES and Pen/Strep. The sample preparation is described in the method section: "For flow cytometry, cells were washed once with PBS containing 2% fetal calf serum (FCS) and resuspended in 20 µl of a 100-fold dilution of allophycocyanin-conjugated anti-murine TCRβ (clone H57-597, eBioscience) in PBS containing 2% FCS. Cells were stained for 20 min on ice and washed twice with PBS containing 2% FCS."

Instrument | Gallios (Beckman Coulter), serial number: AU01404

Software | FlowJo 9.3.2 and 10 (Tree Star)

Cell population abundance | The cells used in this study are derived from the TCRa- TCRb- Jurkat cell line.

Gating strategy | Samples were gated on living cells, FSC/SSC gates are shown for figure 7, boundaries between TCRβ positive and negative cell populations were set according to untransduced TCRa- TCRb- Jurkat cells stained with anti-TCRβ-APC antibodies that define TCRβ negative cells

☒ Tick this box to confirm that a figure exemplifying the gating strategy is provided in the Supplementary Information.
